# Impact of Single-Pulse, Low-Intensity Laser Post-Processing on Structure and Activity of Mesostructured Cobalt Oxide for the Oxygen Evolution Reaction

**DOI:** 10.1021/acsami.1c08034

**Published:** 2021-07-29

**Authors:** Eko Budiyanto, Swen Zerebecki, Claudia Weidenthaler, Tim Kox, Stephane Kenmoe, Eckhard Spohr, Serena DeBeer, Olaf Rüdiger, Sven Reichenberger, Stephan Barcikowski, Harun Tüysüz

**Affiliations:** ‡ Max-Planck-Institut für Kohlenforschung, Kaiser-Wilhelm-Platz 1, Mülheim an der Ruhr 45470, Germany; § Technical Chemistry I and Center of Nanointegration Duisburg-Essen (CENIDE), University of Duisburg-Essen, Universitätsstraße 7, Essen, North Rhine-Westphalia 45141, Germany; ⊥ Department of Theoretical Chemistry, University of Duisburg-Essen, Universitätsstraße 2, Essen, North Rhine-Westphalia 45141, Germany; || Max Planck Institute for Chemical Energy Conversion, Stiftstrasse 34-36, Mülheim an der Ruhr 45470, Germany

**Keywords:** reactive laser processing, defect engineering, oxygen evolution reaction, cobalt oxide, electrocatalyst, X-ray spectroscopy

## Abstract

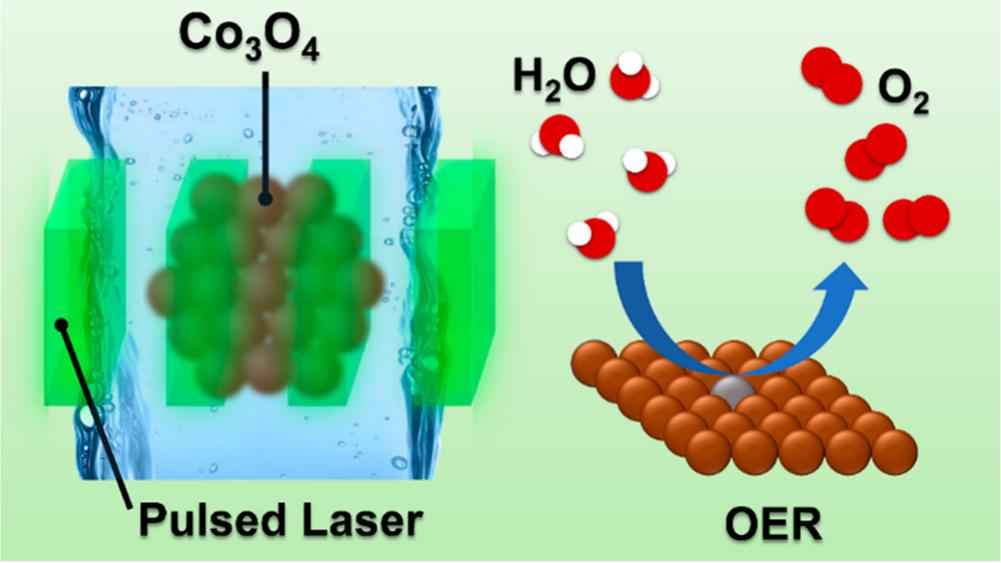

Herein, we report nanosecond, single-pulse laser post-processing (PLPP) in a liquid flat jet with precise control of the applied laser intensity to tune structure, defect sites, and the oxygen evolution reaction (OER) activity of mesostructured Co_3_O_4_. High-resolution X-ray diffraction (XRD), Raman, and X-ray photoelectron spectroscopy (XPS) are consistent with the formation of cobalt vacancies at tetrahedral sites and an increase in the lattice parameter of Co_3_O_4_ after the laser treatment. X-ray absorption spectroscopy (XAS) and X-ray emission spectroscopy (XES) further reveal increased disorder in the structure and a slight decrease in the average oxidation state of the cobalt oxide. Molecular dynamics simulation confirms the surface restructuring upon laser post-treatment on Co_3_O_4_. Importantly, the defect-induced PLPP was shown to lower the charge transfer resistance and boost the oxygen evolution activity of Co_3_O_4_. For the optimized sample, a 2-fold increment of current density at 1.7 V vs RHE is obtained and the overpotential at 10 mA/cm^2^ decreases remarkably from 405 to 357 mV compared to pristine Co_3_O_4_. Post-mortem characterization reveals that the material retains its activity, morphology, and phase structure after a prolonged stability test.

## Introduction

With the depletion of fossil-based energy sources, and the projected incrementally increasing demands for sustainable and CO_2_ emission-free energy, electrochemical water splitting will play a prominent role in the conversion of renewable electricity to “green” hydrogen.^1^ However, the efficiency of water electrolysis is still limited by a sluggish anodic reaction, namely, the oxygen evolution reaction (OER), which requires four electron transfer steps and a relatively high overpotential.^2^ Moreover, the practical application of electrolyzers requires an electrocatalyst with long-term stability and an overpotential below 300 mV to reach current densities of 10 mA/cm^2^, which is analogous to a solar-to-fuel conversion device with 10% efficiency.^3^ So far, iridium- and ruthenium-based oxides have been used as benchmark catalysts for OER because of their high catalytic activity despite the finite supply and scarcity of these noble metals.^4,5^

In the past decade, many studies have been carried out to substitute noble metal electrocatalysts with low-cost first-row transition metal-based oxides.^6−9^ Owing to the moderate OER catalytic activity, tunable defect sites, and good stability due to the unique reversible surface amorphization properties, cobalt oxide (Co_3_O_4_) has gained interest as a promising candidate for water electrolysis.^9−11^ However, the use of bulk Co_3_O_4_ as a substitute for noble metals catalyst is limited because of its low conductivity. Size- and shape-controlled cobalt oxide could bring advantages to boost OER activity by increasing the surface area, as well as the number of exposed active sites.^12,13^ Various synthetic approaches, such as coprecipitation,^14^ solvothermal,^15^ hydrothermal,^16^ solid-state chemical method,^17^ and hard-templating,^18^ are widely used to synthesize nanoparticles and mesostructured cobalt oxides. Recently, we also reported a facile synthesis protocol for mesostructured cobalt oxide by utilizing biomass (tea and coffee-waste) as a hard template.^6,18^ This sustainable and reproducible synthesis approach could be utilized as a toolbox for further post-processing experiments.

Post-processing methods, by either increasing the surface area or engineering structural defects on cation and anion sites, have proven to be useful means to improve the conductivity, as well as increase the active site populations on the catalyst’s surface. A recent study by Yu et al. shows a simple way to synthesize 5 nm cobalt oxide particles with a selective acid leaching method.^19^ The post-treated material shows a superior OER activity compared to the pristine oxides due to a larger surface area and more exposed active sites. Similarly, high-surface-area hierarchical mesoporous Co_3_O_4_ could be fabricated by using Mg as the sacrificial agent.^20^ The post-processed material shows a 2-fold enhancement of the photochemical oxygen evolution activity compared to the pristine sample. Sacrificial agent leaching, surface treatment, doping, and thermal treatments under oxidative or reductive atmospheres are the most common methods of catalyst post-processing.^19−21^ However, they often result in simultaneous materials property changes like particle growth, surface restructuring, defect induction/curing, or introduction of heteroatoms (doping) or impurities. In comparison to these methods, pulsed laser post-processing represents a novel and scalable approach, which precisely tunes catalysts regarding their defect density while mainly retaining their textural parameters, phase structure, and phase purity. This is especially useful for heterogeneous catalysis research where correlation to material properties (phase structure, surface area, and defect density) is still the major tool for investigation. Surfactant-free sub-5 nm cobalt oxide also has been synthesized by pulsed-laser ablation in liquid (PLAL).^22,23^ This synthesis method yields structural defects and high turnover frequency (TOF) per surface site of Co for OER. In general, laser fragmentation or laser ablation of oxides are beneficial for inducing the formation of oxygen defects.^24^ The oxygen defects in cobalt oxides are likely to increase the affinity of the defect sites for water molecules and boost OER activity by facilitating adsorption sites for the dissociative hydroxyl group.^23,25^

In our previous work, mesostructured sub-5 nm cobalt oxide particles were synthesized in a flowing water system by pulsed laser post-processing (PLPP) in liquid with a picosecond laser. A significant increase in surface area was generated in the laser-treated CoO along with structural defects at tetrahedral coordinated Co^2+^ of evolved Co_3_O_4_ after laser treatment. Both factors lead to a superior catalytic activity over pristine cobalt oxides.^12^ Thus far, the pulsed laser was focused on a cylindrical liquid jet to achieve particle fragmentation, which requires very high laser intensity (∼7 × 10^14^ W m^2^). Hence, multiple superimposed material transformation processes (e.g., defect induction,^26^ electron ejection,^27^ phase decomposition,^28^ melting,^29^ evaporation/fragmentation^30^) can occur during the high-intensity laser irradiation. Consequently, the resulting rather broad property distribution makes the distinct correlation of induced defects, surface area, and phase composition to the OER activity complicated.

**Scheme 1:**
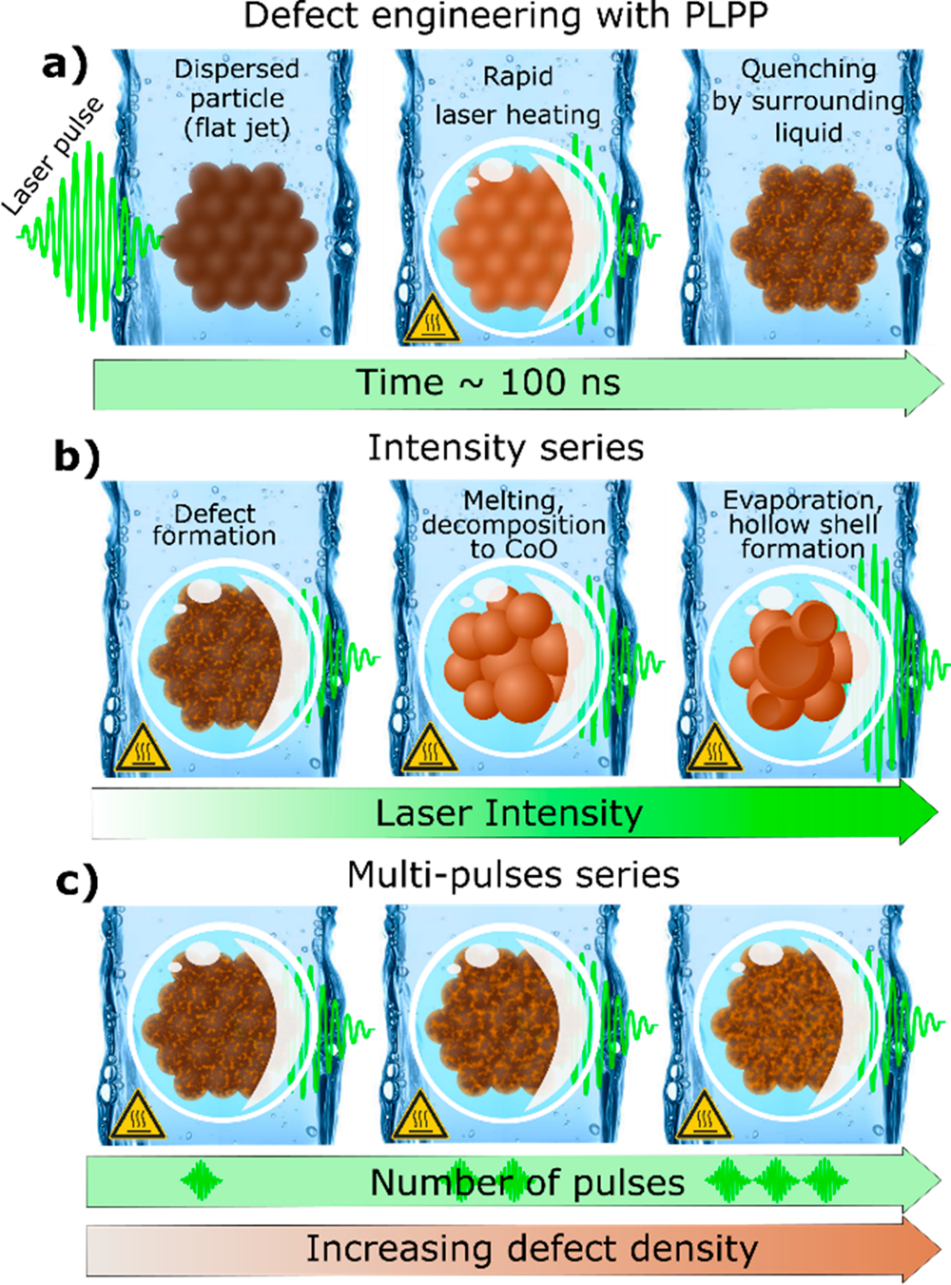
Illustration of Nanosecond Pulsed Laser Post-Processing and its Working Principle (a) Laser irradiation of the particle dispersed in a liquid (water) flat jet causes photothermal heating of the particle which generates a vapor bubble. After the collapse of the vapor bubble, the particle temperature is rapidly quenched which causes freezing of the thermally-induced defects within the structure. (b) Variation in the laser intensity allows the determination of the intensity threshold for the different laser-induced processes of defect-induction, melting, and reduction/decomposition. (c) Increasing the number of applied pulses with intensities below the melting/decomposition threshold causes an increase in induced defect density.

Recently, we developed a setup utilizing a liquid flat jet, which allows for the precise control of the applied laser intensity to the particle dispersion.^31^ Hence, an in-depth study of the laser irradiation with defined and moderate laser intensities (max. 6.2 × 10^11^ W m^–2^) is required to identify the intensity threshold of the different laser-induced processes to circumvent the previously discussed issues. In the present study, instead of picosecond laser pulses (as used in our previous laser fragmentation study on cobalt oxides^12^), nanosecond laser pulses were utilized to exclude potential material changes caused by electron ejection. With nanosecond (ns) laser pulses, the expected main cause of laser-induced material alteration is via photothermal heating,^30^ which is depicted schematically in Scheme 1 a. For Co_3_O_4_, the 532 nm laser pulses are expected to excite the electrons (potentially inducing electron transfer from O^2–^ to the Co^3+^) in the Co_3_O_4_ particles, which compared to pulse duration (7 ns) instantly (∼0.5 ps) transfers their energy to the lattice, causing a rapid temperature increase.^32^ If the temperature of the particle surrounding water is above the spinodal temperature of 277 °C, a vapor bubble forms with an expected lifespan of ∼20–200 ns.^33^ This bubble formation isolates the particles thermally from the liquid. Plech et al. recently showed via in situ XAS measurements (during picosecond laser fragmentation) that the bubble signal decays within 30 ns and inferred that when bubble has vanished, the transiently hot nanomaterial cools down within 1–2 ns.^34^ Hence, with the collapse of the nanobubble, the particle temperature is quenched back to ambient temperatures, freezing the transient structure. Accordingly, these quick temperature changes can potentially induce lattice disorder and allow for defect engineering. At the same time, by applying low laser intensity (low particle temperatures), processes that affect the total surface area (melting, evaporation, fragmentation) or the phase composition change can potentially be avoided. This allows the separation of laser-induced defect generation from processes that influence the surface area (via particle size change) or the phase composition and therefore allows for the specific correlation of the generated defects to the OER activity.

To verify the hypothesis, we engineered defect density and structural disorder on mesostructured Co_3_O_4_ in two sets of experiments with controlled laser intensity (as depicted in Scheme 1b) and pulse number variation (as depicted in Scheme 1c). The intensity series allowed for the identification of the intensity-threshold for Co_3_O_4_ decomposition and particle melting while increasing the number of applied laser pulses below this threshold allows for the specific increase in defect density. Note that the liquid jet′s flow velocity together with the adjustment of the laser repetition rate allows precise control over the number of laser pulses per liquid volume, in increments of single pulses per particle dispersed in that volume. We found that the irradiation at >1 × 10^11^ W m^–2^ induces particles melting as well as the evolution of a CoO minor phase from Co_3_O_4_ decomposition. The increment of laser intensity induces structural disorder in Co_3_O_4_, resulting in the formation of CoO and amorphous cobalt hydroxide phases that significantly affect the OER performance.

## Experimental Section

### Synthesis of Coffee-Templated Cobalt Oxide

Coffee-templated mesostructured Co_3_O_4_ was synthesized via the hard templating method following the reported protocol.^18^ Briefly, Co(NO_3_)_3_·6H_2_O (Sigma-Aldrich, ACS reagent grade 99.5% purity) precursor solution in distilled water was impregnated into the coffee template. After direct combustion at 400 °C for 4 h (ramping rate of 2 °C/min), the obtained material was then leached with 0.1 M HCl solution, rinsed with distilled water, and dried at 80 °C overnight.

### Continuous Pulse Laser Post-processing (PLPP)

PLPP experiments were conducted with the previously reported flat jet setup^31^ and a 532 nm pulsed laser (IS400-1-L, SHG, Edgewave) at a pulse duration of ∼7 ns and maximum pulse energy of ∼10 mJ (∼50 W at a 5 kHz repetition rate). The laser beam profile was measured with a Beamage 4 M camera before every experiment. The original laser beam profile was truncated with two mechanical slits to gain a uniform beam profile. To allow single pulse per particle conditions (i.e., pulses per volume element = PPV) the time between subsequent laser pulses (repetition rate of the laser) was set to match the calculated residence time of a volume element inside the laser spot (0.2 ms = 5 kHz). The overall flow rate through the flat jet nozzle was 150 mL/min. The flow rate of the irradiated liquid was around 13 mL/min.

### Material Characterization

Wide-angle powder XRD was measured with a Stoe theta/theta diffractometer operating in reflection mode with Cu Kα_1,2_ radiation X-ray source (λ: 1.5406 Å) using a step size of 0.02° 2θ. The instrument was equipped with an energy discriminating detector. The XRD patterns for qualitative phase analysis of sample deposited on carbon fiber paper were collected on a Stoe STADI P transmission diffractometer using Mo Kα radiation (0.7093 Å). The instrument is equipped with a primary Ge (111) monochromator (Mo Kα_1_) and a position-sensitive Mythen1K detector. Phase identification was based on the comparison of experimental data with the PDF-2 ICCD database.^35^ Nitrogen physisorption measurement was carried out using 3Flex Micromeritics at 77 K. The sample was degassed under vacuum at 120 °C for 10 h before the measurements. Brunauer–Emmett–Teller (BET) surface area was calculated in the relative pressure range (*p*/*p*°) of 0.06 to 0.3. Pore size distribution was calculated with the Barrett, Joyner, and Halenda (BJH) method applied to the desorption branch of isotherm. Transmission electron microscopy (TEM) micrographs, as well as high-resolution images, were taken with 200 keV acceleration voltage of cold field-emission gun (FEG) Hitachi HF2000 electron microscope. Secondary electron (SE) imaging was performed using a Cs-corrected Hitachi HD-2700 dedicated scanning-transmission electron microscope (STEM) equipped with a cold field-emission gun. The sample was dry-casted on a copper grid (400 mesh) covered with a lacey carbon film. Raman spectra were recorded with a Renishaw InVia Raman microscope with a 532 nm laser wavelength. X-ray photoelectron spectroscopy (XPS) measurements were carried out with a PHI 5000 Versaprobe II, utilizing a monochromatic aluminum anode with a Kα line at 1486.6 eV, a spot size of 100 μm, a hemispherical analyzer (with an angle of 45° between the surface of the sample and the analyzer), and dual beam charge neutralization. All of the XPS spectra were referenced to the C 1s peak. *Casa* XPS was used to analyze the spectra. Synchrotron X-ray diffraction data were collected at the high-resolution powder diffraction beamline (P02.1) at PETRA III (DESY). Samples were filled into 0.5 mm glass capillaries and mounted on the sample spinner. Data were collected at 60 keV (λ = 0.20723 Å) with a PerkinElmer XRD1621 area detector. Rietveld refinements were performed with the program package *TOPAS* V6, Bruker AXS GmbH, Karlsruhe, Germany.^36^

### X-ray Spectroscopy

Co Kβ XES was collected at beamline I20-scanning at the Diamond Light Source (3 GeV, 300 mA). A Si(111) double crystal monochromator was used for energy selection of the incident beam, and a rhodium-coated mirror was used for harmonic rejection, delivering a flux of ∼4 × 10^12^ photons/s at the sample position. X-rays were focused to achieve an approximate beam size of 100 × 300 μm^2^ (*V* × *H*). A Johann-type XES spectrometer was used with two Ge(444) crystals aligned by setting the maximum of Kβ emission line of a Co foil to 7649.1 eV. The incident energy was calibrated by setting the first inflection point of the Co XAS spectra to 7709.0 eV for a Co foil. Data was collected at room temperature. All samples for XAS data collection were prepared diluted to approximately 10 mass % Co in dry boron nitride, pressed into a 0.5 mm PEEK sample holder, and sealed between 38 μm Kapton tape. Samples for XES were packed in the same manner but measured without dilution. Co Kβ XES spectra were collected from 7620 to 7670 eV, with a step size of 0.2 eV. Co K-edge XAS and EXAFS spectra were collected in fluorescence mode using a four-element Vortex Si-drift detector. EXAFS data processed with the Athena program and analyzed using Artemis with the *IFEFFIT* software package.

### Electrochemical Measurements

Electrochemical oxygen evolution reaction (OER) was carried out in an alkaline condition (1 M KOH as the electrolyte solution) and evaluated by using a BioLogic SP-150 potentiostat and a rotating disc electrode (model AFMSRCE, PINE Research Instrumentation) with a three-electrode system. The system was constructed using a Pt wire as the counter electrode, hydrogen reference electrode (HydroFlex, Gaskatel) as the reference electrode, and a sample deposited on glassy carbon (5 mm diameter, 0.196 cm^2^ geometric surface area) as the working electrode. Before deposition, glassy carbon electrodes were polished with an alumina suspension (1 and 0.05 μm, Allied High-Tech Products, Inc.), and 5.25 μL of as-prepared catalyst ink (consisting of the homogeneous dispersion of 4.8 mg of electrocatalyst in 0.75 mL of Mili-Q water (18.2 MΩ.cm), 0.25 mL isopropanol, and 50 μL Nafion-117 (Sigma-Aldrich)) was then drop-casted onto the glassy carbon electrode and dried in Ar flow under the lamp illumination. The catalyst loading was calculated to 0.12 mg/cm^2^. Ar flow was bubbled into the electrolyte solution to remove the oxygen before each measurement. The temperature of the electrochemical cell was kept at 25 °C by using a thermostat. The measurements were done with 2000 rpm rotation speed of the working electrode and the *i*R-drop was compensated at 85%. Linear scan voltammetry (LSV) curves were recorded within 0.7–1.7 V vs RHE potential range and scan rate of 10 mV/s. Cyclic voltammetry (CV) was measured within the potential range of 0.7 to 1.6 V vs RHE and scan rate of 50 mV/s. Chronopotentiometry was recorded at 10 mA/cm^2^ to examine the stability of the electrocatalyst over time. Electron Impedance Spectroscopy (EIS) was carried out in a frequency range of 100 mHz to 100 kHz and 5 mV amplitude. The anodic polarization potential was kept at 1.6 V vs RHE during EIS measurement. The as-recorded Nyquist plot was then fitted to the simulated data using the Z-Fit feature in *EC-Lab* software. The double-layer capacitance (*C*_dl_) was measured with the cyclic voltammetry method by varying scan rates at the non-Faradaic region. *C*_dl_ was calculated as the half-slope of scan rates against the capacitive current (Δ*j* = *j*_anode_ – *j*_cathode_). Electrochemical surface area (ECSA) was then estimated by dividing the double layer capacitance with specific capacitance (*C*_s_). The *C*_s_ value for 1 M KOH electrolyte is 0.04 mF/cm^2^.^37^ Inductively coupled plasma-optical emission spectrometry **(**ICP-OES) measurement was carried out with SPECTROGREEN instrument, and the electrolyte solution was sampled from the electrochemical cell before and after chronopotentiometry.

## Results and Discussion

The initial mesostructured Co_3_O_4_ for PLPP experiments was synthesized via a hard-templating approach by using coffee waste as a sustainable template.^18^ The characterization of the as-synthesized Co_3_O_4_ is presented in Figure S1a–d. Briefly, a large amount of mesostructured Co_3_O_4_ with sheetlike morphology and BET surface area of 70 m^2^ g^–1^ is obtained by simple impregnation and combustion of the coffee template. The higher magnification on the TEM micrograph (Figure S1b) shows that the sheet-like morphology consists of sintered nanoparticles with an average particle size of around 7 nm. The as-synthesized material is phase-pure Co_3_O_4_ as proven by well-defined XRD reflections (Figure S1c) that match the cubic structure of the reference (PDF-2 00–42–1467). Then, pulse laser post-processing (PLPP) with nanosecond laser pulses and variation in laser intensities (0.5, 1.2, 2.3, and 6.2 × 10^11^ W m^–2^, labeled as PLPP0.5, PLPP1.2, PLPP2.3, and PLPP6.2, respectively) were applied under single pulse per volume element conditions to the aqueous Co_3_O_4_ dispersion in the liquid flat jet. The previously described rapid temperature changes under pulsed laser irradiation are expected to induce structural changes in the material.^12,31,38^

Powder XRD measurements were conducted to investigate the crystalline phases and potential structural and/or compositional transformations of the Co_3_O_4_ samples. Powder XRD patterns of Co_3_O_4_ samples before and after PLPP are shown in Figure S2. There are neither observable phase changes nor significant broadening of reflections of the PLPP0.5 sample. Conversely, the Co_3_O_4_/CoO double-phase starts to emerge at PLPP1.2 and becomes more pronounced for higher laser intensities. This indicates that the peak particle temperature during laser irradiation with ∼1.2 × 10^11^ W m^–2^ already reaches above 800 °C, where thermal decomposition of Co_3_O_4_ to CoO is expected to emerge.^39^ The appearance of CoO from laser-induced thermal decomposition of cobalt-based spinel has also been reported in our previous works, however, at three orders of magnitude higher intensities (1 × 10^14^ W m^–2^) to achieve effective laser fragmentation.^12,38^ Hence, for laser-induced fragmentation experiments, strong phase decomposition seems to be unavoidable for Co_3_O_4_, unlike for the milder excitation conditions addressed in the present study.

**Figure 1:**
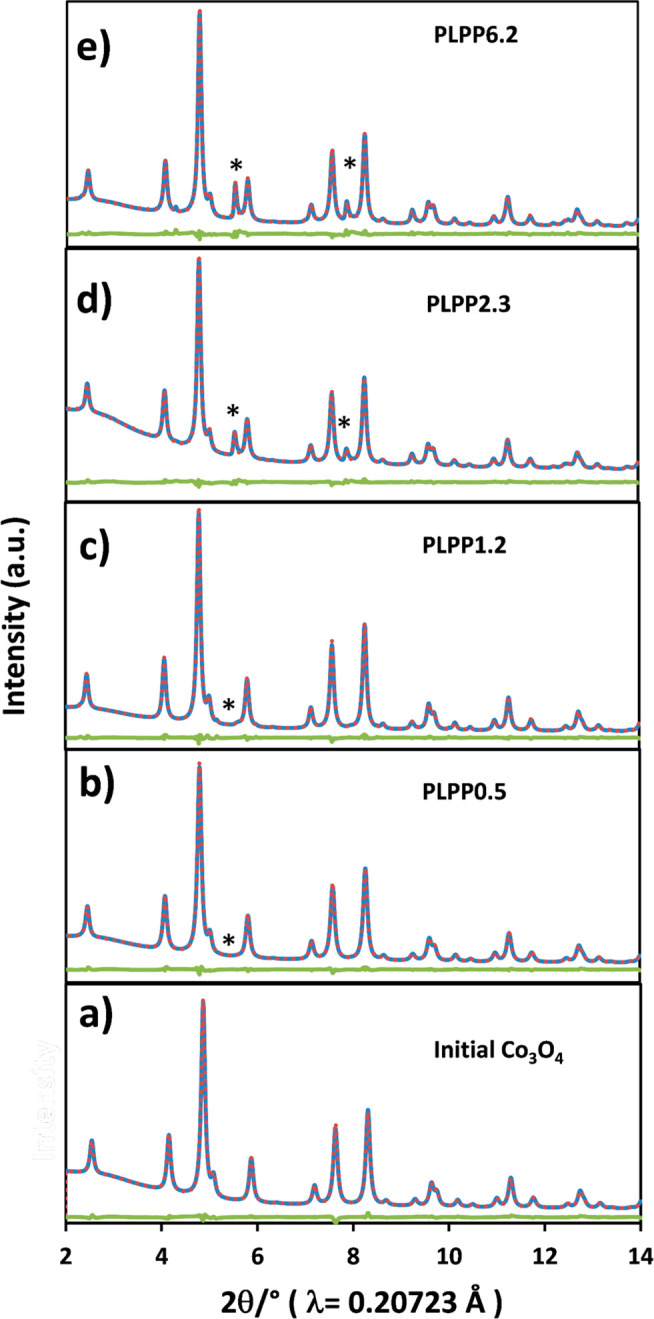
Rietveld refinement of high-resolution XRD patterns (a) Initial Co_3_O_4_, (b) PLPP0.5, (c) PLPP1.2, (d) PLPP2.3, and (e) PLPP6.2 with single-pulse. Asterisk denotes the reflections of CoO. Blue line, measured data; red dots, calculated data; and green lines, difference curves.

To shed light on the crystalline structure disorders after PLPP and precisely quantify the emerged CoO phase, we then measured high-resolution XRD with synchrotron radiation. The Rietveld refinement of high-resolution XRD patterns is shown in Figure 1a–e and the summarized data are shown in Table S1. At laser intensities above ∼1 × 10^11^ W m^–2^, the laser treatment induces the formation of CoO with up to 11 wt % at the highest laser intensity of 6.2 × 10^11^ W m^–2^. Additionally, Rietveld refinement reveals that lower occupancy of cobalt on the tetrahedral sites (Co^T^) of the spinel phase is observed in some PLPP samples, indicating that cobalt in tetrahedral sites is more easily ejected from the spinel’s lattice during laser irradiation, creating cation vacancies.

Zhang et al. indicated the formation of defects in tetrahedral sites of Co_3_O_4_ is more preferable due to a lower defect formation energy in comparison with the octahedral counterparts.^40^ These cobalt vacancies at tetrahedral sites are responsible for the subtle lattice expansion due to the elongation of Co–Co bonds. The increase in lattice parameter also could be related to the formation of oxygen vacancies; however, this cannot be determined without refinement of neutron diffraction data.^41^

Nitrogen physisorption measurements were carried out to examine the textural parameters (surface area, pore structure, and pore size distribution) of initial and PLPP samples. All samples have a type IV adsorption–desorption isotherm that is typical for mesoporous materials, and the H3 hysteresis loop depicts the assemblage of slit-shaped pores (Figure S3a, b).^42^ The initial Co_3_O_4_ BET surface area of 70 m^2^/g remained constant for PLPP0.5 and PLPP1.2, whereas a clear decrease of up to 12% was found for PLPP2.3 and PLPP6.2 (Figure S3c), likely due to particle melting accompanied by particle growth. This indicates that the temperature of the particles during PLPP with high laser intensity exceeds the melting point of Co_3_O_4_ (∼900 °C).^43^

**Figure 2:**
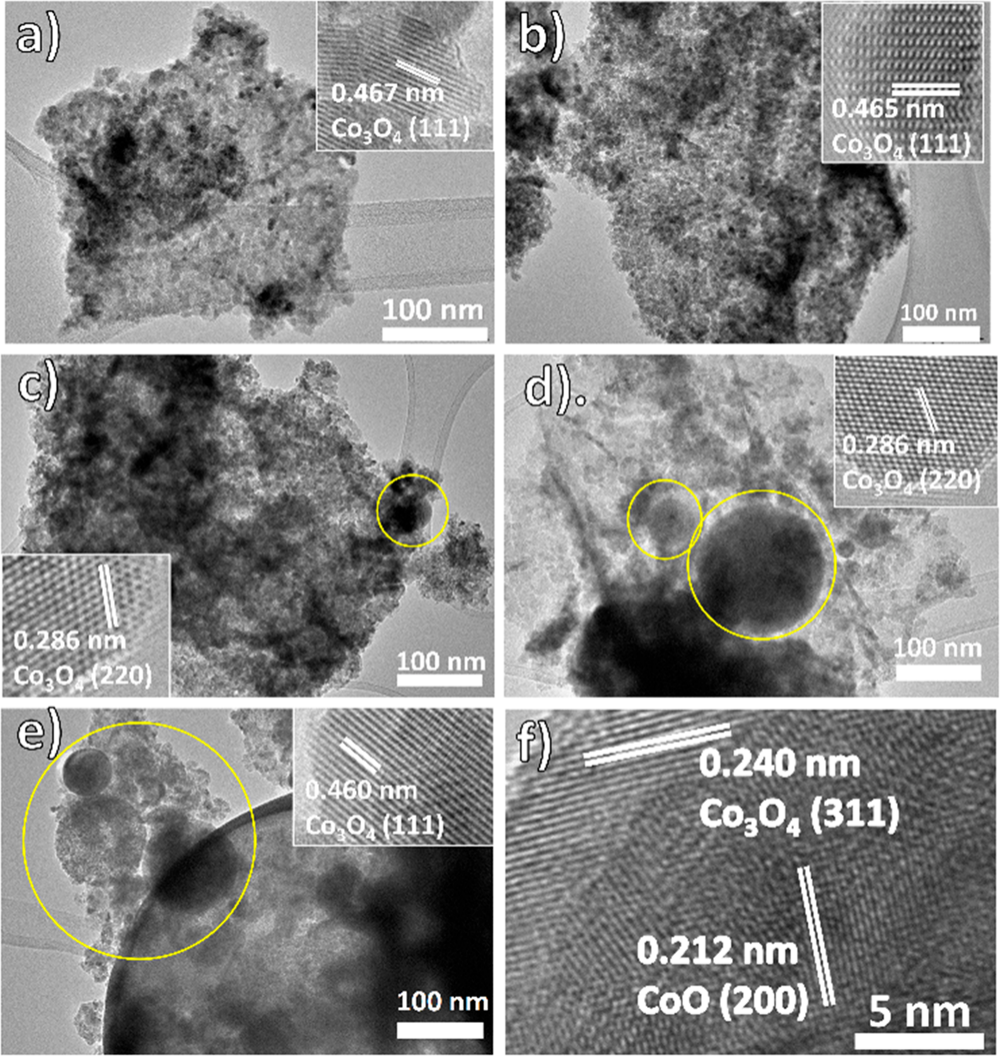
TEM micrographs with the inset HR-TEM of single-pulse PLPP samples, (a) Pristine Co_3_O_4_, (b) PLPP0.5, (c) PLPP1.2, (d) PLPP2.3, and (e) PLPP6.2, and (f) HR-TEM image of melted particles in PLPP6.2. Lattice fringes are referred to as PDF-200-42-1467 (Co_3_O_4_) and PDF-200-48-1719 (CoO). Yellow circles indicate laser-induced particles melting.

A transmission electron microscopy (TEM) study was further performed to verify the former hypothesis. As shown in Figure 2a, b, pristine Co_3_O_4_, and PLPP0.5 have similar sheetlike morphology. However, significant particle growth by laser-induced particle melting becomes very pronounced at higher laser intensities (Figure 2c–e). This led to the decrease in BET surface area in PLPP samples that are irradiated with high laser intensities, as previously mentioned. High-resolution TEM imaging depicts that crystallinity is maintained after PLPP, even at the highest applied laser intensity. The lattice fringes measurement at a thin section of melted particles (Figure 2f) also reveals the coexistence of the Co_3_O_4_ phase and domains of a CoO phase in the near-surface region for the samples treated with the highest laser intensities.

Apart from particle melting, the occasional formation of amorphous and hollow-sphere particles is observable at PLPP6.2 (see Figure S4a–f). The formation of hollow shell particles under pulsed laser irradiation was also previously reported in the literature for Fe_3_O_4_ and Co_3_O_4_ by Ishikawa et al.^44^ They proposed that the hollow particles form during pulsed laser melting of aggregated particles in liquid after single-pulse irradiation, causing a void to be trapped inside the molten particles. It has been also shown that hollow gold nanoparticles could be produced by single laser pulse-induced evaporation of particles in the liquid.^45,46^ The thin hollow shell particles observed after high laser intensity treatment are mainly crystalline sub-2 nm particles (see Figure S4c, d). Similarly, STEM micrographs on selected hollow structure (see Figure S4e, f) shows that the thin hollow structure consists of aggregates of very small particles. Therefore, the hollow structures may be generated from an explosive formation of a gas bubble inside the laser-induced molten particle aggregates. These gas bubbles could originate from laser-induced evaporation of Co_3_O_4_ and the subsequent decomposition to CoO, which generates O_2_ or photothermal water splitting that is also known to result from laser ablation.^47^ These thin mesoporous particle-shell formations may be beneficial for avoiding the formation of bulk particles and keeping the BET surface area loss to only 12% of initial cobalt oxide even at the highest laser intensity.

Raman spectra were recorded to study the vibrational modes and electronic structure change on PLPP samples (Figure S5). During the measurement, the Raman laser power was kept low (∼0.8 mW) to avoid the oxidation of CoO to Co_3_O_4_ in the PLPP samples.^48^ Five well-resolved bands are observed in all samples, corresponding to A_1g_, E_g_, and three F_2g_ phonon modes generated from lattice vibration of the spinel phase.^49^ One weak phonon mode of CoO is also observed as a hump at around 550 cm^–1^ for PLPP2.3 and PLPP6.2 (Figure S5a). The second phonon mode that presents at 690 cm^–1^ was barely observed because of the low percentage of CoO and overlap with the A_1g_ band from the spinel phase.^48^ An expanded view of the A_1g_ band (Figure S5b) shows the asymmetrical broadening in PLPP samples. The asymmetric broadening could be correlated to the lattice disorder due to the introduction of defects and an increase of lattice parameters, in agreement with the results of the aforementioned Rietveld refinement.^50^ It also indicates a lower crystallization degree and change in electronic structure due to the increasing amount of amorphous phase and cobalt at a lower oxidation state (e.g., octahedrally coordinated Co^2+^ in CoO rock salt structure or amorphous Co(OH)_2_) existence after laser treatment.^51−53^

X-ray photoelectron spectroscopy (XPS) data were then recorded to gain information on the oxidation state of cobalt at the surface region. Figure S5c shows Co 2p spectra characteristic for Co_3_O_4_. The clear increase in intensity at around 785–786 eV could be ascribed to the satellite peak characteristic for Co^2+^ in CoO or Co(OH)_2_. This indicates an increase in Co in the oxidation state of 2+ on the surface after PLPP with increasing laser intensities.^54−56^ Considering the cobalt deficiency on the tetrahedral sites of Co_3_O_4_ after PLPP, the increase in Co^2+^ at the surface might be attributed to the formation of CoO or amorphous Co(OH)_2_ domains at the surface as confirmed by HR-TEM. According to the gathered data, PLPP appears to increase the defect density of Co_3_O_4_, even though a minor phase change into CoO is inevitable for high laser intensities due to thermal decomposition.

**Figure 3:**
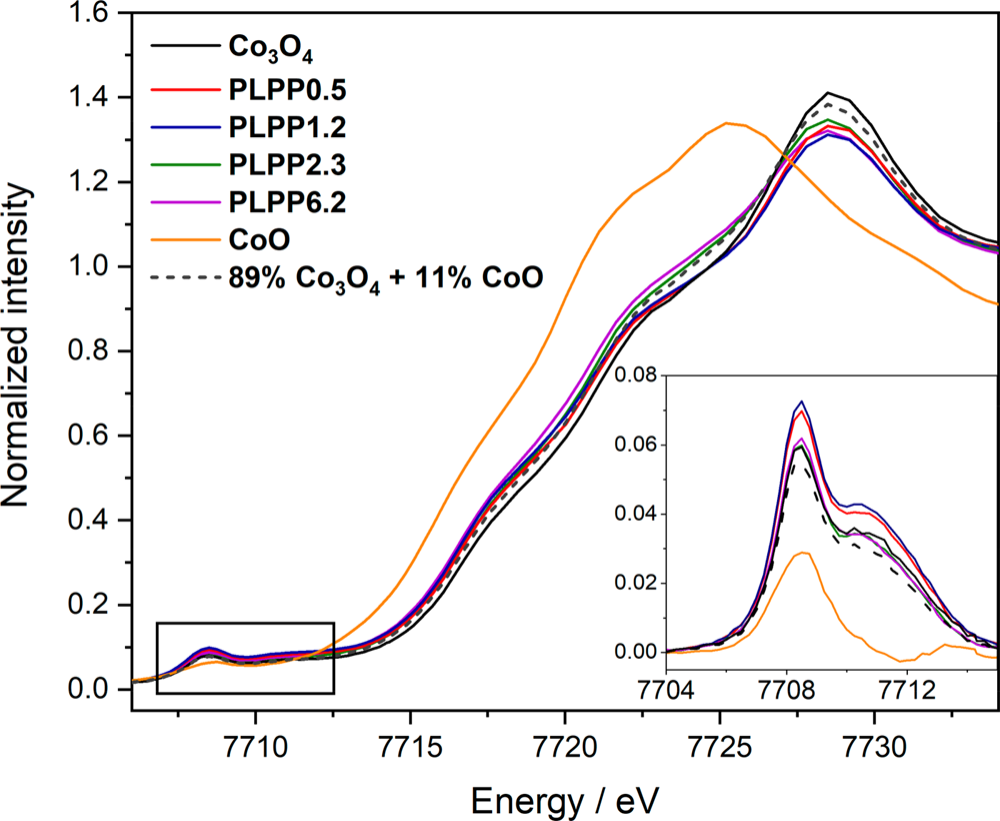
Co K-edge partial fluorescence yield (PFY) XAS spectra of CoO, Co_3_O_4_, and the PLPP samples. The inset shows in detail the baseline-corrected pre-edge region.

The Co K-edge XAS (Figure 3) shows a small shift of the rising edge to lower energies for all the PLPP samples suggesting a slight decrease in the average overall oxidation state for these samples. The pre-edge, originating from the dipole-allowed 1s → 3d transition show some small differences among the samples (Figure 3, inset). Interestingly, the PLPP0.5 and PLPP1.2 samples have a higher XAS intensity than untreated Co_3_O_4_, whereas the higher laser intensity samples, PLPP2.3 and PLPP6.2, have pre-edges that are essentially identical to untreated Co_3_O_4_. XRD indicates that CoO forms only in samples that were irradiated at the higher laser intensities (e.g., PLPP2.3 and PLPP6.2, with a maximum of 11% CoO for the PLPP6.2 sample). Hence, we attempted to model the XAS data with a mixture of 11% CoO and 89% Co_3_O_4_ spectra (dashed line in Figure 3). In all cases, the edge shifts more than what would be expected from such a combination, and importantly we observe a similar edge shift for all PLPP samples and not only for the ones where XRD detected CoO. Additionally, the pre-edge intensity cannot be modeled just by mixing CoO and Co_3_O_4_, because adding CoO always lowers the pre-edge intensity (in CoO, cobalt has octahedral coordination, and therefore a weaker pre-edge).

Although XRD suggests the presence of a CoO phase in the stronger laser-excited PLPP2.3 and PLPP6.2 samples, the pre-edge does not lose intensity, and hence there must be an additional source of pre-edge intensity. A possible explanation for this discrepancy between XRD and XAS would be that the laser-treated samples have a small percentage of oxygen-deficient Co sites, small enough that it does not affect the long-range structural ordering and therefore is not reflected in the XRD pattern.^23^ This would explain the pre-edge intensities and the edge positions. A higher coordination number on an absorber has been found to shift the edge to higher energy due to electrostatic destabilization of the 4p orbitals.^57^ Additionally, the strong pulsed laser-induced photothermal heating of the Co_3_O_4_ particles in the water environment is expected to induce surface restructuring and formation of amorphous phase (Figure S4b), likely attributed to cobalt hydroxide. Solvation of surface Co_3_O_4_ by water is known to induce minor restructuring and hydroxylation, which improves OER activity.^10,58,59^ The elevated particle temperature during PLPP is expected to increase this hydroxylation resulting most likely in the formation of α-Co(OH)_2_ phase that has been reported to be composed of a portion of Co^2+^ in tetrahedral geometry that increases the overall pre-edge intensity in XAS spectra and a weak octahedral contribution.^60^ We hypothesize that this amorphous α-Co(OH)_2_ could not be detected by XRD because the method is sensitive only to crystalline phase. This thus could explain the mismatch in XAS spectra modeling with the mixing of 11% CoO and 89% Co_3_O_4_.

The Co K-edge EXAFS spectra were fitted with a simple four-path model (Figure S6), which yields acceptable fittings for all the spinel samples. The fitting deteriorates for the high laser intensity samples. Although the Co–O distances do not significantly change among the series of spinel samples, the laser-treated samples show increased Co–Co distances and Debye–Waller factor (Table S2), indicating a more disordered sample. This is consistent with the subtle lattice parameter expansion as calculated by Rietveld refinement. The Kβ XES (Figure S7) shows minimal variation upon laser treatment; however, at high laser power, there is a slight increase in the Kβ′ intensity, which suggests an increased average spin of the sample, consistent with more Co^2+^ from a CoO or Co(OH)_2_ phases.^61^

To investigate the dependence of pulse laser intensities to the structural changes, we performed a calculation of the maximum particle temperature during pulsed laser irradiation (Figure S8). It was calculated that the particle temperature could reach ∼800 K for PLPP0.5, ∼2600 K for PLP1.2, ∼5600 K for PLPP2.3, and ∼16 000 K for PLPP6.2 (see the description in Figure S8). Note that the temperature calculation does not include a phase change or decomposition. However, the calculated temperatures correlate sufficiently with the observation of the first phase decomposition at PLPP1.2 considering the decomposition temperature of Co_3_O_4_ at 1100 K.^39^ They also indicate that evaporation of the Co_3_O_4_ or CoO might play a role in the formation of the observed hollow spheres (Figure 2d, e) at respective laser intensity used to prepare PLPP2.3 and PLPP6.2. To study the surface restructuring at mild laser excitation conditions (below the laser intensity thresholds given in Figure S8) before the hollow sphere formation and phase decomposition occurs, we performed additional molecular dynamics simulations of a Co_3_O_4_/water interface at 300, 700, and 1000 K over a total simulation time of 10 ps (Figure S9a–c). In the given time scale, the increase in temperature results in a stronger interaction between the reconstructed Co_3_O_4_ (001) surface and the water/hydroxides molecules resulting in more Co^2+^ being pushed up from the surface into tetrahedral coordination, as can be seen from the atom density profiles in Figure S9c (an average of two coordination sites were taken by water/hydroxides and two from the lattice oxygen). This is in line with the increase in overall pre-edge intensity in XAS spectra after laser treatment, indicating the increase of Co^2+^ at tetrahedral geometry. Further, these surface hydroxide/water structures are possibly potential precursors for the formation of the amorphous cobalt hydroxide after laser irradiation and might play a crucial role in the formation of the active site during OER.

**Figure 4:**
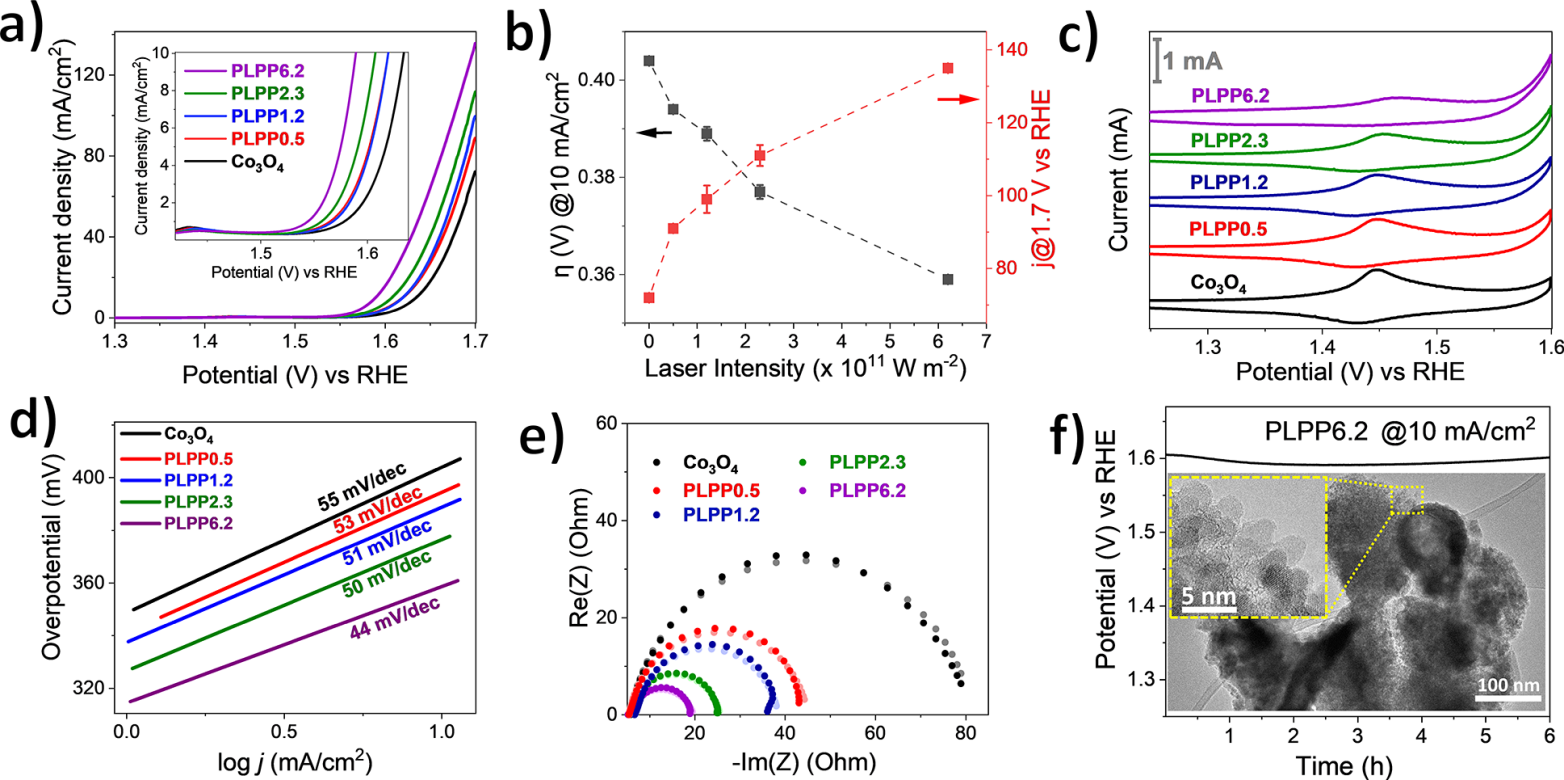
OER data of PLPP intensity series: (a) stabilized LSV curves on OER in 1 M KOH solution with the inset of the enlarged region of initial OER, (b) summary of overpotential (η) at 10 mA/cm^2^ geometric surface area and current density (*j*) at 1.7 V vs RHE, (c) overlaid cyclic voltammograms at 50th cycle with the scan rate of 50 mV/s, the actual current is depicted by the scale bar, (d) Tafel slope, (e) experimental Nyquist plot (dark dots) with corresponding fitted data (pale dots), and (f) stability test measured at 10 mA/cm^2^ with the corresponding TEM micrograph of the catalyst scratched from the working electrode after stability tests.

To examine the effect of laser intensity on the catalytic activity of PLPP samples, we tested the materials for electrochemical oxygen evolution reaction (OER). As shown by stabilized LSV curves in Figure 4a and the measured overpotential at 10 mA/cm^2^ and current density at 1.7 V vs RHE in Figure 4b, PLPP treatment is a very effective method for enhancing the OER activity of pristine Co_3_O_4_. The overpotential at 10 mA/cm^2^ significantly decreased from 405 mV to 360 mV for PLPP6.2, while the current density at 1.7 V vs RHE increased by up to 2-fold, despite the decrease in BET surface area compared to initial Co_3_O_4_ (Figure S3c). The OER enhancement due to the high-laser intensity treatment (PLPP2.3 and PLPP6.2) could be attributed to structural disorder and defects or the formation of highly active amorphous Co(OH)_2_ and CoO phases after the laser treatment.^21,62^ The OER enhancement could also be observed on the lowest laser intensity (PLPP0.5), which contains less than 1% of the CoO phase. This confirms that the formation of defects or structural disorder in Co_3_O_4_ also plays a key role to enhance OER activity.

Cyclic voltammogram (CV) is a useful technique to evaluate the oxidation–reduction behavior at the surface of electrocatalysts during applying potential bias. As shown in Figure 4c, the anodic peak around 1.45 V vs RHE is attributed to the oxidation of high valent cobalt (Co^3+^ to Co^4+^) and is widely proposed as the prominent factor for the increased reaction rate of OER.^63−65^ In contrast with OER activity, the gradual decrease of the anodic peak at 1.45 V vs RHE with increasing laser intensity indicates that the oxidation of Co^3+^ to Co^4+^ is not the sole factor for OER activity in PLPP samples. Recent studies reported that the reducibility of Co^3+^–O in CoO_*x*_(OH)_*y*_ intermediate also plays a prominent role in catalyzing water oxidation and the reducibility of Co^3+^–O could be estimated by comparing the current at the first and second cathodic peaks (C1/C2 ratio).^11,66^Figure S10 shows a comparison of CV between the most active sample (PLPP6.2) and pristine Co_3_O_4_. The PLPP sample has a pronounced higher C1/C2 ratio compared to the pristine Co_3_O_4_, proving the increase of Co^3+^–O reducibility by PLPP. The increasing of Co^3+^–O reducibility in the OER state could be attributed to the coexistence of Co_3_O_4_, CoO, and cobalt hydroxide phase on the surface of samples before potential bias. Co^3+^–O from CoO_*x*_(OH)_*y*_ intermediate species (as a result of oxidation of CoO and cobalt hydroxide during application of potential bias) seems to be more easily reduced compared to Co^3+^–O in the CoO_*x*_(OH)_*y*_ intermediate from pristine Co_3_O_4_ oxidation.^11^ The easily reducible Co^3+^–O may be beneficial for inducing fast OER by promoting μ_2_-OH bridges between high-spin cobalt ions in the CoO_*x*_(OH)_*y*_ intermediate and enhancing electron transfer.^11^

Tafel slopes were calculated from the stabilized LSV to investigate the OER kinetics and plotted in Figure 4d. The Tafel slope value of 55 mV/dec for pristine Co_3_O_4_ is identical to the reported Tafel slope in analogous works.^12,40,61,64^ The Tafel slopes of the PLPP samples decrease with increasing laser intensities, indicating that laser post-processing may enhance the OER kinetics. The enhancement of OER kinetics is likely attributed to the increased reducibility of the Co^3+^–O (as observed from cyclic voltammetry) and the pronounced formation of a CoO second phase after laser treatment. More importantly, the preferential defect formation on tetrahedral sites (as confirmed by high-resolution XRD data) additionally plays a key role to induce fast OER kinetics. DFT calculations performed by Zhang et al.^40^ indicate that cobalt vacancies at tetrahedral sites induce structural disorder and increases charge density around the conducting band edge. This is consistent with the increase in Co–Co distances and Debye–Waller factors from our EXAFS data (Figure S6 and Table S2). As a result, faster charge transfer results when applying potential bias and increases the OER kinetics of the PLPP samples.

A complementary experiment with electrochemical impedance spectroscopy (EIS) was carried out to measure charge transfer resistance (*R*_ct_) and verify the enhancement of electron transfer on PLPP samples (Figure 4e). The obtained Nyquist plot is fitted to simplified Randles model (*R*_Ω_)(*R*_ct_*Q*_dl_) as presented in Figure S11 and the EIS data fitting for the laser intensities series are summarized in Table S3.^19,37,67^ A unified electrolyte solution resistance of 5–6 Ω is found for all samples. In agreement with the previous discussion on cyclic voltammetry and the Tafel slope, the laser-irradiated samples have a lower charge transfer resistance compared to initial Co_3_O_4_. This illustrates the advantage of PLPP treatment of Co_3_O_4_ for inducing electronic alterations that favor rapid electron transfer without the formation of fragmented particles nor increase in BET surface area.

Chronopotentiometry at 10 mA/cm^2^ was conducted on the most active PLPP samples deposited on a glassy carbon electrode to examine the stability of electrocatalyst during the continuous applied constant current as seen in Figure 4f. The sample shows plausible stability for up to 6 h of measurement with slight deactivation after 4 h that is attributed to the instability and irreversible oxidation of CoO domain to Co_3_O_4_ phase.^10,11^ Post-mortem TEM and HR-TEM show that the overall morphology of initial materials and small aggregate of particles around the hollow structure was maintained after the stability test. Detailed post-mortem HR-TEM (see Figure S12) reveals that the Co_3_O_4_ phase is mostly found in the near-surface of particles after application of long-term potential bias.

To further verify the influence of structural defects on mesostructured Co_3_O_4_ toward electrochemical OER enhancement, we then prepared a set of PLPP samples with multipulse (continuous) PLPP at low laser intensity (0.5 × 10^11^ W m^–2^) where no phase-change was observed in Figure 2 (PLPP0.5). This method aimed to synthesize defect-rich mesostructured Co_3_O_4_ while minimizing the evolution of the CoO phase and morphology changes from laser melting. The initial mesostructured Co_3_O_4_ was treated with fixed laser intensity and the variation pulses (1, 2, 3, and 4). As shown in the XRD pattern (Figure S13a), there is no pronounced reflection corresponding to a CoO phase that emerged up to two pulses. However, a small amount of CoO starts to evolve after three pulses. The Co 2p scan collected by XPS (Figure S13b) shows satellite peaks typical for Co_3_O_4_ for all pulse series samples without any noticeable increase in the satellite peak at 786.1 eV, corresponding to additional Co^2+^ from CoO.^54−56^ In turn, Raman spectra show a clear difference after multipulse laser irradiation (Figure S13c). Five well-resolved bands corresponding to A_1g_, E_g_, and three F_2g_ phonon modes are observable for pristine Co_3_O_4_. The F_2g_(1) band becomes less pronounced with an increasing number of laser pulses while an additional underlying broad Raman band seems to increase the overall Raman band in the region of the F_2g_(1). This may be attributed to the asymmetric broadening of A_1g_ (Figure S13d) upon the laser treatment. This asymmetric broadening of the A_1g_ band was also observed in laser intensity series samples (Figure S5), indicating that lattice disorder also exists in the PLPP multipulse series.

N_2_ adsorption isotherms of multipulses series samples are shown in Figure S14a–c. There is no pronounced change in adsorption–desorption isotherms and pore size distributions indicating the overall pore structures of the Co_3_O_4_ are maintained after multipulse, low-intensity PLPP. However, the BET surface area of the samples decreased after 3 and 4 pulses. TEM micrographs (Figure S15a, b) show only a small number of laser-induced melted particles melting in samples after 1 and 2 pulses. In contrast, the formation of occasional hollow structures is seen in samples after 3 and 4 pulses (Figure S15c, d). Besides, vacancy sites were observed in HR-TEM (Figure S15d), indicating the formation of point defects after PLPP.

**Figure 5:**
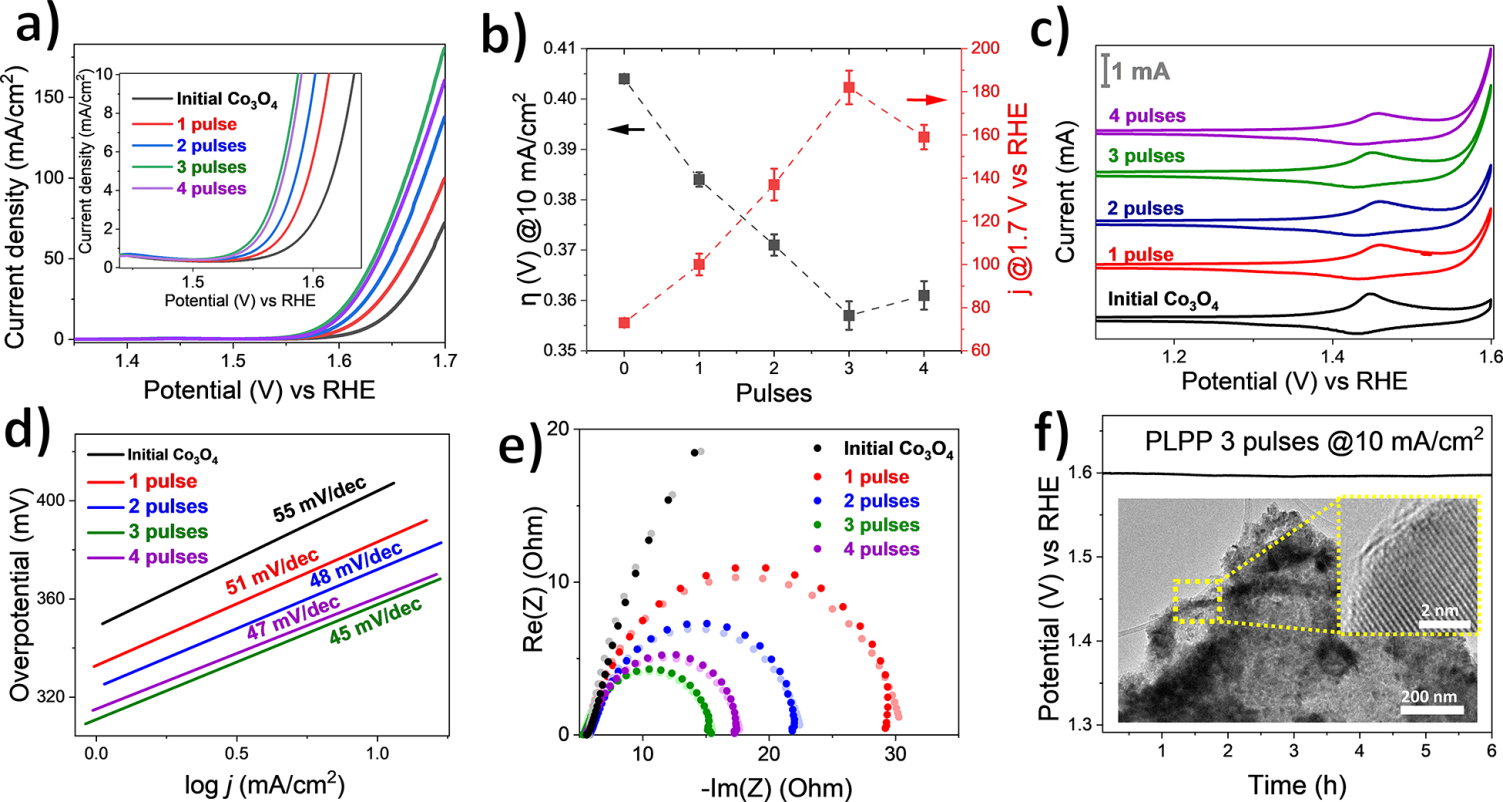
OER data of PLPP multipulse series: (a) stabilized LSV curves on OER in 1 M KOH solution with the inset of the enlarged region of initial OER, (b) summary of overpotential (η) at 10 mA/cm^2^ geometric surface area and current density (*j*) at 1.7 V vs RHE, (c) overlaid cyclic voltammograms at 50th cycle with the scan rate of 50 mV/s, the actual current is depicted by the scale bar, (d) Tafel slope, (e) experimental Nyquist plot (dark dots) with corresponding fitted data (pale dots), and (f) stability test measured at 10 mA/cm^2^ with the corresponding post-mortem TEM and HR-TEM micrograph of the catalyst scratched from working electrode.

OER screening was also conducted on this sample series to examine the influence of pulses number on its catalytic activity (Figure 5a–f). The LSV curves and its summary clearly show an increase in OER activity in the multipulse series. The optimum OER activity is achieved at three pulses that has comparable OER activity to the best sample from the intensity series that was treated with 1 pulse and 10 times higher intensity (6.2 × 10^11^ W m^–2^). The optimized electrocatalyst has a comparable electrocatalytic OER activity in comparison with the recently reported analogous works and benchmarked cobalt oxide (see Table S4). For the sample treated with 3 pulses and low intensity (0.5 × 10^11^ W m^–2^), less CoO phase was present compared to the 11 wt % CoO in the single pulse and high-intensity case (PLPP6.2). This indicates that the formation of defects on Co_3_O_4_ itself outweighs the beneficial influence of the CoO phase on the OER activity. After four pulses, OER-activity slightly decreased in comparison to three pulses, indicating that the maximum sustainable defect density was already reached after three laser pulses. The decrease in activity after three laser pulses appears to be linked to the slight decrease in BET surface area and formation of larger hollow spheres and aggregates (see Figured S14c and S15d).

No significant differences in the CV curves of the multipulse series were observed (Figure 5c). This could be ascribed to a similar oxidation state in the near-surface region as confirmed by XPS. Double-layer capacitance (*C*_dl_) and electrochemical surface area (ECSA) were then calculated by varying scan rate of CV measurements at non-Faradaic region (Figure S16a–e).^37,68^ Both values increased with the number of low-intensity laser pulses and reach a maximum at three pulses (Figure S16f, g). This indicates that the density of active sites increases by performing multipulses PLPP on mesostructured Co_3_O_4_ samples up to saturation and removal, possibly due to purification and segregation effects that occur at high defect density and ongoing laser treatment.

In summary, the increase of ECSA without the subsequent increase of BET surface area indicates the formation of defects in the crystalline lattice, which then potentially decrease the H_2_O molecules’ adsorption energy onto the catalyst’s surface.^69^ ECSA normalized LSV curves (Figure S17) show that the increasing pulse number enhances the OER intrinsic activity that is caused by the increase in defect density. Similarly, the three pulses sample shows the fastest OER kinetics (Figure 5d) and least charge transfer resistance (Figure 5e and summarized fitted data in Table S5).

The stability test was carried out on a three pulse sample with 6 h of continuously applied bias at 10 mA/cm^2^ (Figure 5f). This sample shows a constant current without an observable deactivation during the chronopotentiometry test. Finally, post-mortem TEM imaging was conducted with the sample scratched from the glassy carbon electrode after the stability test. The sample maintains its morphology after the stability test, but TEM imaging reveals the formation of amorphous phase artifacts on the near-surface region due to the expected phase transformation from Co_3_O_4_ to amorphous CoO_*x*_(OH)_*y*_ during applying potential bias.^10,70^

A post-mortem study was performed to further examine the possible change in the catalyst’s structure after OER. For this purpose, the sample treated with three laser pulses was deposited onto the carbon fiber paper (CFP) and the stability test at 10 mA/cm^2^ was extended up to 12 h. The sample shows good stability without OER activity decrease up to 12 h (Figure S18a, b). In addition, there is no detectable cobalt leaching into the electrolyte solution after the stability test as measured by inductively coupled plasma-optical emission spectrometry (ICP-OES). The material retains its sheet-like morphology without observable agglomeration (Figure S18c). Raman spectroscopy and XRD measurements were then carried out to see the possible change in the bulk region of material after the stability test. Both samples before and after the stability test show five unshifted Raman bands that corresponding to the lattice vibrations of the spinel structure (Figure S18d).^49^ Likewise, similar XRD patterns are observed for samples before and after OER (Figure S18e). These indicate that the bulk region of the material mainly remains after OER. The assignment of the CoO minor phase is not possible in the XRD pattern because of the overlap of reflection between the main peak of CoO and that of carbon fiber paper. XPS measurement was performed to probe the structural change in the near-surface region after the OER. The high-resolution Co 2p and O 1s scans are shown in Figure S19a, b and the data fitting is summarized in Table S6. The Co 2p scan (Figure S19a) shows a decrease in satellite peaks at 787.1 eV that can be ascribed to the irreversible oxidation of Co^2+^ from the amorphous surface cobalt hydroxide phase into Co^3+^ in the CoOOH or spinel phase after applied potential bias.^11,54,55^ In addition, the O 1s scan (Figure S19b) reveals the increases in relative intensity ratio at 531.0 and 532.0 eV that correspond to lattice O in hydroxide and adsorbed −OH, respectively.^55,56^ This indicates the existence of a thin layer of amorphous CoOOH on the catalyst surface as an artifact of surface amorphization during OER as observed by the HR-TEM image in the inset of Figure S18c.^10^

## Conclusions

The nanosecond continuous-flow pulsed-laser post-processing (PLPP) was applied to induce structural defects in mesostructured Co_3_O_4_. Two sets of experiments were conducted with varying laser intensities and pulse numbers. In the single-pulse experiments, significant photothermal melting and decomposition were observed only at laser intensities above 1 × 10^11^ W m^–2^, whereas phase purity was maintained at lower laser intensity. Rietveld refinement of high-resolution XRD data, Raman, XPS, and XAS/XES spectra confirmed the formation of CoO as well as structural disorder and increasing lattice parameter with the increase in applied laser intensity. Molecular dynamics simulation on Co_3_O_4_/water interface further confirmed the surface restructuring upon laser post-treatment of Co_3_O_4_ nanoparticles. The low-intensity (0.5 × 10^11^ W m^–2^), single-pulse PLPP sample performance confirms that the formation of defects or structural disorder in Co_3_O_4_ is key to enhance OER activity, with the preferential defect formation on tetrahedral sites additionally inducing fast OER kinetics. The low laser intensity was then selected for multipulse PLPP experiments and it was found that treating Co_3_O_4_ with three low-intensity laser pulses resulted in the optimized OER electrocatalyst. The density of active sites increased with the number of PLPP pulses reaching saturation at the pulses. This treatment caused structural disorder within mesostructured Co_3_O_4_, whereas phase purity and morphology were maintained, also showing a good durability within 6 h of continuous applied bias. The laser-induced structural disorder of cobalt sites was identified to boost OER activities by increasing Co^3+^-O reducibility, decreasing charge transfer resistance, and increasing the number of active sites. Post-mortem characterization revealed that sample treated with three low-intensity laser pulses maintained its OER activity, morphology, and phase structure after a prolonged stability test without detectable cobalt leaching into the electrolyte solution.

## Supporting Information Available

Characterization of the coffee templated pristine Co_3_O_4_; wide-angle XRD of fluence series; Rietveld refinement; textural parameters of fluence series; TEM micrographs of fluence series; Raman and XPS spectra of fluence series; Fourier transform, *k*^3^-weighted χ(*k*), and fitting parameters of Co K-edge EXAFS spectra; Kβ X-ray emission spectra; calculated maximal particle temperature dependence on laser intensities; molecular dynamics simulations of a Co_3_O_4_/water system at different temperatures; cyclic voltammetry; Randles circuit; EIS fitting of fluence series; post-mortem HR-TEM image; wide-angle XRD, Raman, and XPS of pulses series; textural parameters of pulses series; TEM images of pulses series; double-layer capacitance and ECSA measurements; ECSA normalized LSV; EIS fitting of pulses series; post-mortem characterization data (PDF)

## Dedication

In memory of Prof. Chia-Kuang (Frank) Tsung.
