# Supplementary material for: Impact of Single-Pulse, Low-Intensity Laser Post-Processing on Structure and Activity of Mesostructured Cobalt Oxide for the Oxygen Evolution Reaction
Source: ACS Appl Mater Interfaces. 2021 Jul 29;13(44):51962–73. doi: 10.1021/acsami.1c08034 (PMC8587604; doi:10.1021/acsami.1c08034)
Supplement: Supplementary file 1 — am1c08034_si_001.pdf [file am1c08034_si_001.pdf]

Supporting Information

Impact of Single-Pulse, Low-Intensity Laser Post-Processing on Structure and Activity of Mesostructured Cobalt Oxide for the Oxygen Evolution Reaction

Eko Budiyanto, [‡†] *Swen Zerebecki,* [‡⊥] *Claudia Weidenthaler,*[†] *Tim Kox,*[#] *Stephane Kenmoe,*[#] *Eckhard Spohr,*[#] *Serena DeBeer,*[§] *Olaf Rüdiger,* [§] *Sven Reichenberger,*[⊥] *Stephan Barcikowski,**[⊥] *and Harun Tüysüz**[†]

† Max-Planck-Institut für Kohlenforschung, Kaiser-Wilhelm-Platz 1, 45470 Mülheim an der Ruhr, Germany

⊥Technical Chemistry I and Center of Nanointegration Duisburg-Essen (CENIDE), University of Duisburg-Essen, Universitätsstraße 7, Essen, North Rhine-Westphalia, 45141, Germany

Department of Theoretical Chemistry, University of Duisburg-Essen, Universitätsstraße 2, Essen, North Rhine-Westphalia, 45141, Germany

§ Max Planck Institute for Chemical Energy Conversion, Stiftstr. 34-36, 45470 Mülheim an der Ruhr, Germany

‡ These authors contributed equally to this work.

*⊥E-mail: stephan.barcikowski@uni-due.de

*†E-mail: tueysuez@kofo.mpg.de

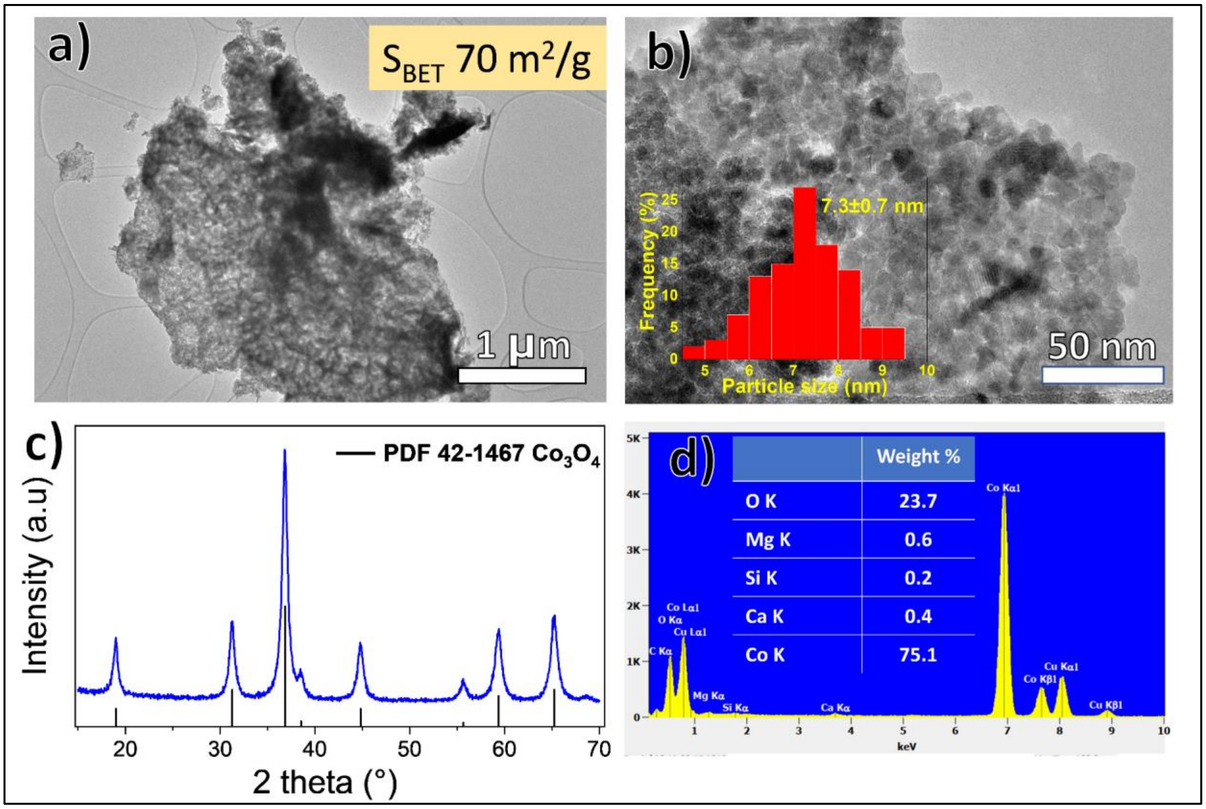

Figure S1. The characterization of coffee-templated Co_3O_4 (a) Low magnification TEM micrograph, (b) high magnification TEM micrograph with its particle size distribution, (c) XRD pattern with PDF-2 00-42-1467 reference, and (d) elemental analysis with EDX spectra. Note: C and Cu EDX spectra (from TEM grid) are excluded from the quantification, Mg and O weight percentages are overestimated in quantification due to the low atomic number.

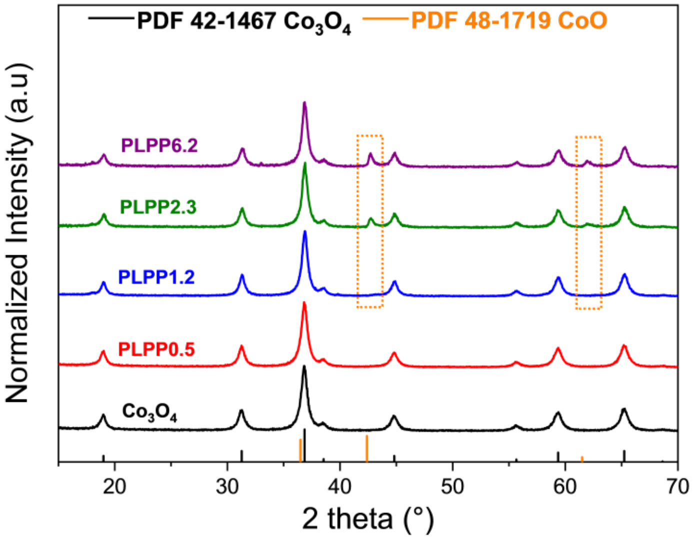

Figure S2. Wide-angle XRD patterns of initial Co_3O_4 and PLPP-treated samples with the variation of laser intensities. The orange-dotted box indicating the evolution of CoO at high laser intensities.

Table S1. Summary of the results obtained ba Rietveld refinement, Co^T denotes cobalt occupancy at tetrahedral sites and Co^O denotes cobalt occupancy at octahedral sites.

Label	Intensity ($x10^{11}$ W m^{-2})	a (Å)	Co^T	Co^O	CoO (wt%)
PLPP6.2	6.2	8.0935(1)	0.92	1.0	11
PLPP2.3	2.3	8.0926(1)	0.94	1.0	8
PLPP1.2	1.2	8.0917(1)	0.92	1.0	1
PLPP0.5	0.5	8.0912(1)	0.92	1.0	<1
Initial Co_3O_4	0	8.0913(1)	0.94	1.0	0

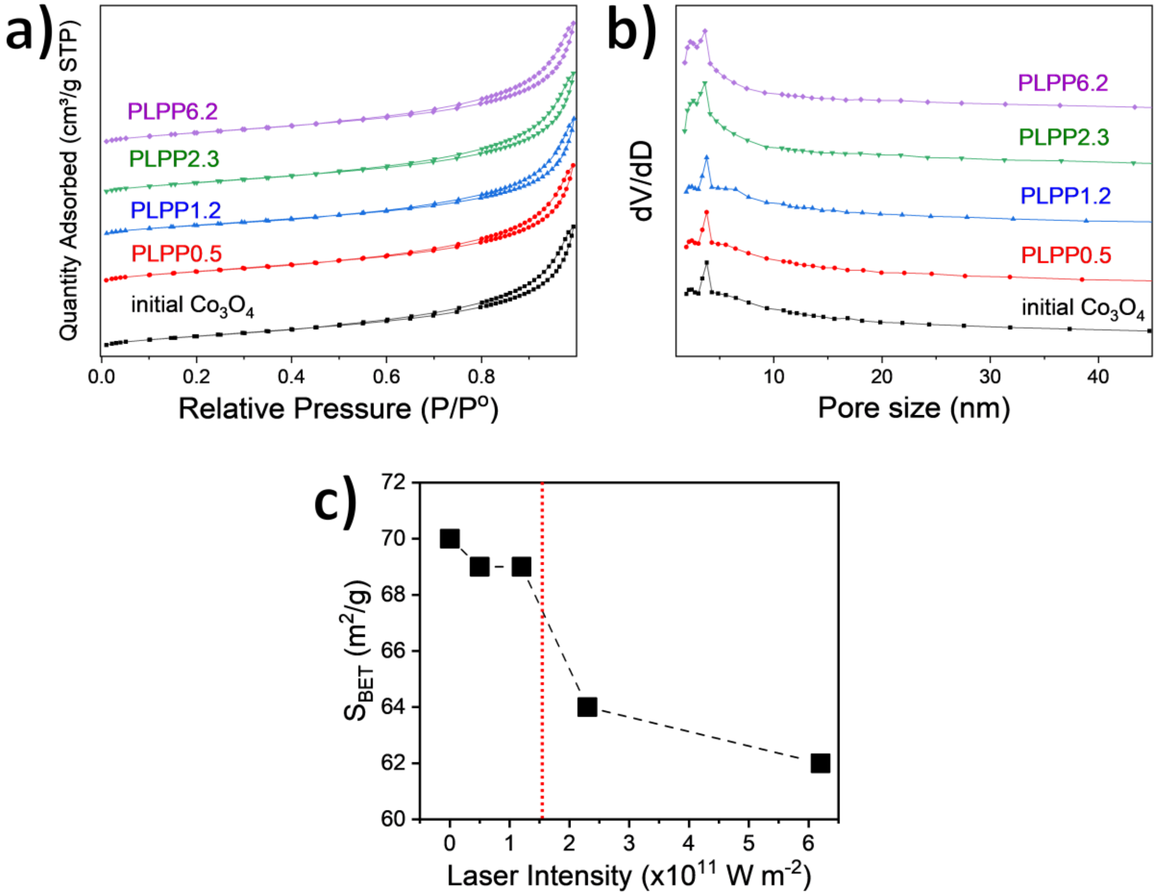

Figure S3. Textural parameters of initial Co_3O_4 and PLPP samples with laser fluences variation, (a) adsorption-desorption hysteresis loop with adsorption branch lies in the lower part and desorption branch lies in the upper part of the hysteresis loop, (b) BJH pore size distribution, and (c) summary of BET surface area.

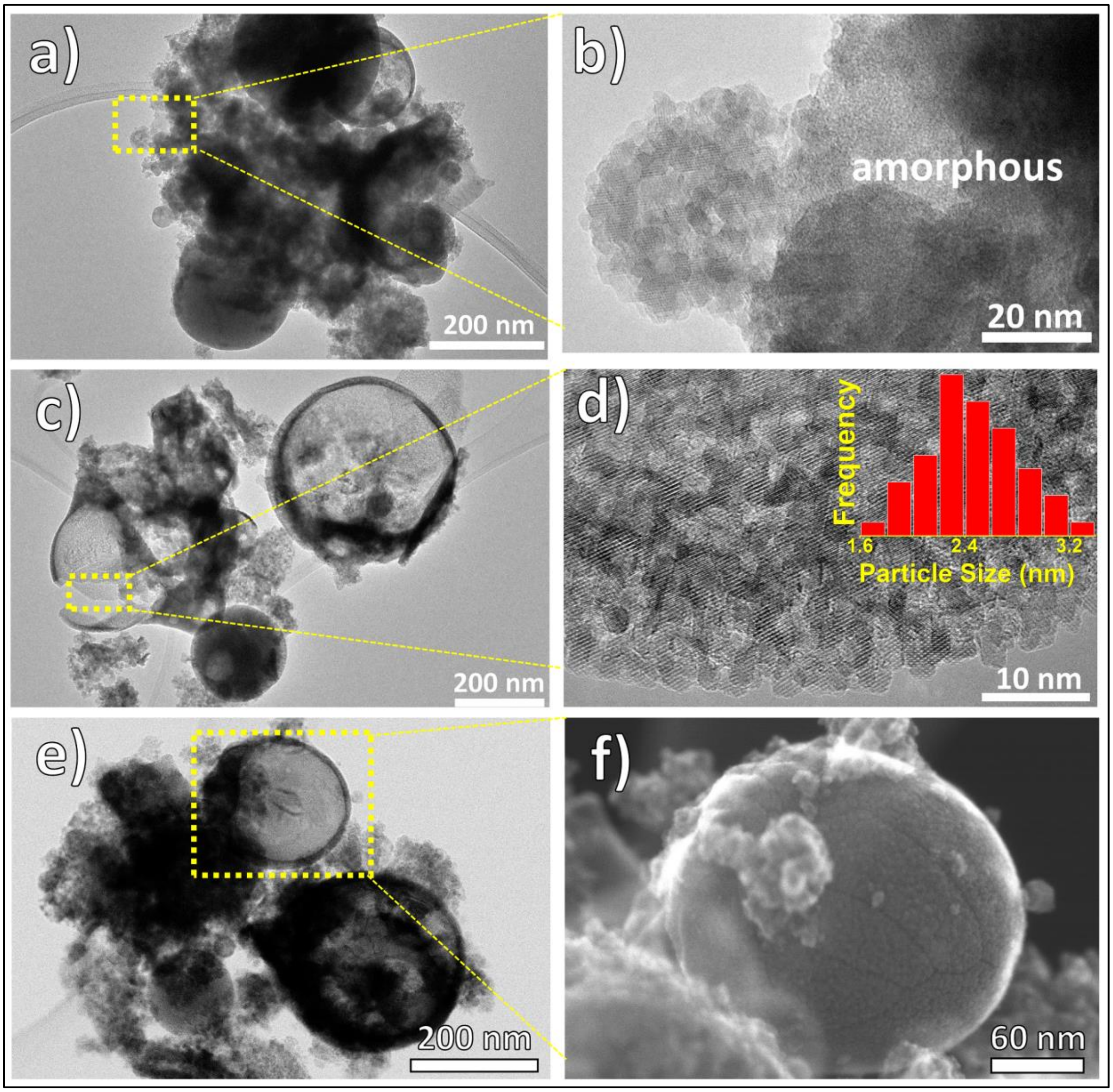

Figure S4. TEM micrographs of the sample irradiated with 6.2 $\cdot 10^{11}$ W m^{-2} laser intensity, depicting the (a) particles melting with corresponding (b) HR-TEM on the amorphous region, (c) thin layer of a hollow sphere with corresponding (d) HR-TEM and particle size distribution, (e) STEM micrograph in transmission mode, and (f) secondary electron imaging on the hollow structure.

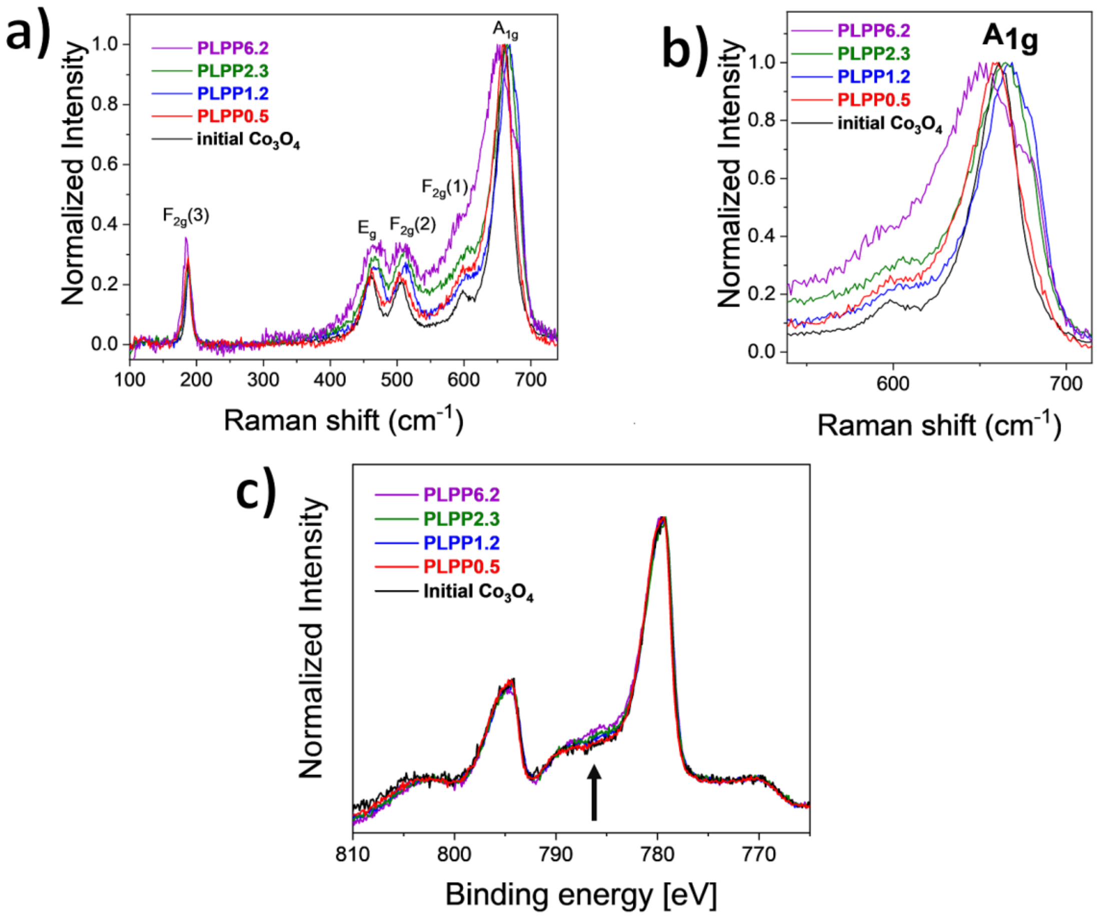

Figure S5. (a) Selected Raman spectra, (b) magnification of A_{1g} phonon mode from Raman spectra, and (c) high resolution Co 2p XPS region of PLPP fluence series samples, the black arrow indicating the increase of the satellite peak corresponding to Co^{2+}.

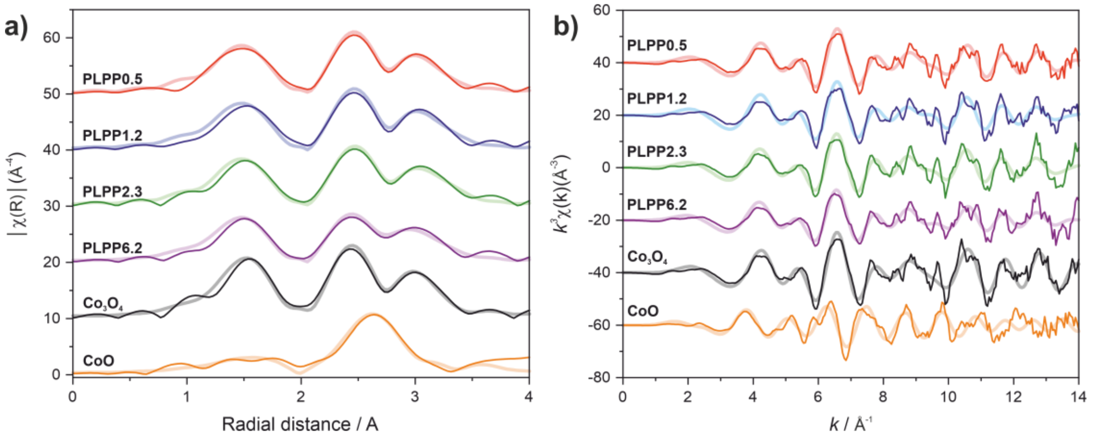

Figure S6. Co K-edge EXAFS spectra of the different samples. a) Real part of the Fourier transform. b) k^3-weighted $\chi(k)$. The thin traces correspond to the experimental data, the thicker faded traces to the fittings.

Table S2. Co K-edge EXAFS fitting parameters for samples.

	Shell	N	σ^2 / Å^2	R / Å	E_0(eV)	R-factor
**Co_3O_4**	$Co^{3+,2+}$-O	**5.3**	0.0043 ±0.0008	1.92±0.01	7720.1	0.017
	Co^{3+}-Co^{3+}	**4**	0.0044±0.006	2.85±0.01		
	Co^{2+}-Co^{3+}	**8**	0.006±0.001	3.37±0.02		
	Co^{2+}-Co^{2+}	**1.3**	0.005±0.005	3.57±0.04		
**CoO**	Co-O	**6**	0.019±0.003	2.11±0.02	7719.9	0.019
	Co-Co	**12**	0.0125±0.0005	3.02±0.01		
	Co-O	**48**	0.0005±0.0016	3.77±0.02		
**PLPP0.5**	$Co^{3+,2+}$-O	**5.3**	0.0062±0.0009	1.91±0.01	7719.52	0.019
	Co^{3+}-Co^{3+}	**4**	0.0058±0.0006	2.85±0.01		
	Co^{2+}-Co^{3+}	**8**	0.009±0.001	3.38±0.02		
	Co^{2+}-Co^{2+}	**1.3**	0.004±0.004	3.62±0.04		
**PLPP1.2**	$Co^{3+,2+}$-O	**5.3**	0.006±0.001	1.92±0.01	7719.71	0.037
	Co^{3+}-Co^{3+}	**4**	0.006±0.001	2.85±0.01		
	Co^{2+}-Co^{3+}	**8**	0.009±0.002	3.39±0.02		
	Co^{2+}-Co^{2+}	**1.3**	0.005±0.005	3.65±0.05		
**PLPP2.3**	$Co^{3+,2+}$-O	**5.3**	0.006±0.001	1.92±0.01	7718.69	0.018
	Co^{3+}-Co^{3+}	**4**	0.0061±0.0007	2.86±0.01		
	Co^{2+}-Co^{3+}	**8**	0.009±0.001	3.38±0.02		
	Co^{2+}-Co^{2+}	**1.3**	0.003±0.003	3.66±0.03		
**PLPP6.2**	$Co^{3+,2+}$-O	**5.3**	0.007±0.001	1.91±0.01	7717.44	0.031
	Co^{3+}-Co^{3+}	**4**	0.008±0.001	2.85±0.02		
	Co^{2+}-Co^{3+}	**8**	0.010±0.002	3.37±0.02		
	Co^{2+}-Co^{2+}	**1.3**	0.004±0.004	3.63±0.04		

N is the coordination number for the absorber-backscatterer pair; R is the average absorber−backscatterer distance; σ^2 is the Debye−Waller factor; the R-factor gives a fractional misfit (not scaled by the data uncertainty) and it is defined by: $R = \frac{\sum_i^{N_{fit}}\left[\chi_i^{measured} - \chi_i^{model}(x)\right]^2}{\sum_i^{N_{fit}}[\chi_i^{measured}]^2}$.

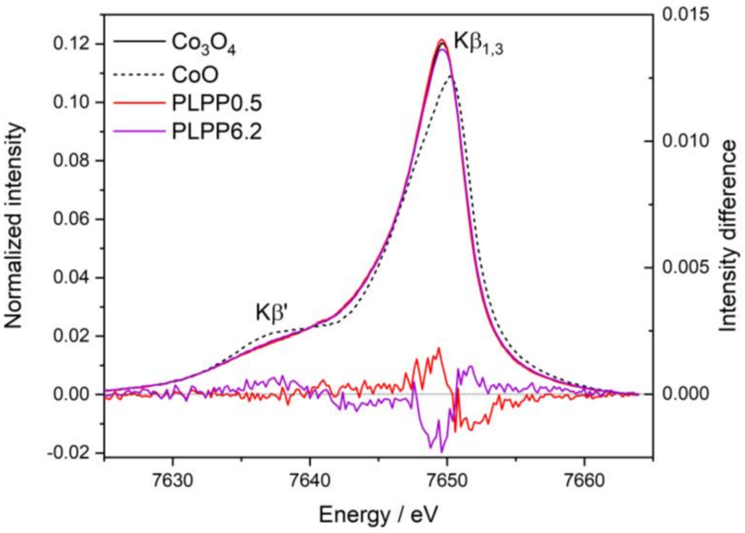

Figure S7. Non-resonant Kβ X-ray emission spectra and difference spectra (PLPP0.5 - Co_3O_4, red; PPLP6.2 - Co_3O_4, purple).

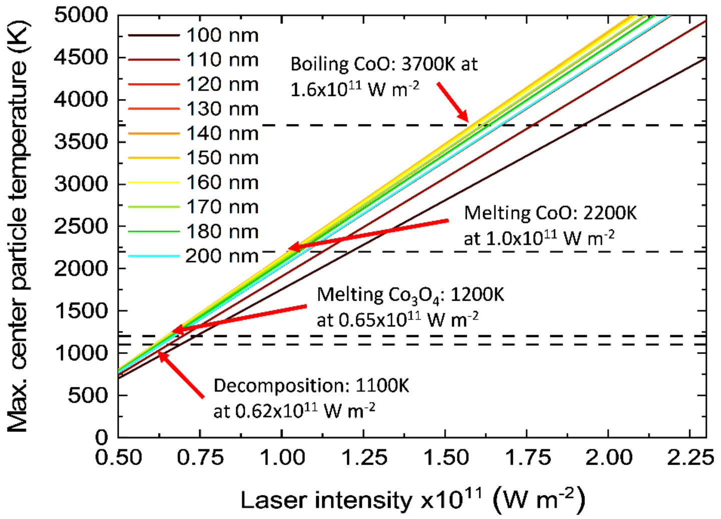

Figure S8. The calculated maximal particle temperatures at the center of different particle diameters in dependence of the pulsed laser intensities.

The melting point data of oxides are retrieved from the physical constant of the inorganic compounds database.[1] The relation was determined with the calculation published by Baffou *et al.*[2, 3] for 7 ns-laser pulses. Utilizing the optical constants of Co_3O_4 published by J. G. Cook and M.P. van der Meer,[4] a heat capacity of 114.44 J/mol K,[5] and a thermal conductivity of 40 W/m/K.[6] The calculated temperatures indicate that particles with a diameter of ~150 nm reach the highest temperature. Since the experimentally used particles have a very non-uniform size in dispersion due to the strong aggregation of their primary particles and the flake-like morphology of these aggregates, the calculation can only be evaluated as a best-case estimation for the particle temperature. Additionally, the heat capacity increases while the thermal conductivity decreases at an increasing temperature which was not considered in this calculation.[6, 7]

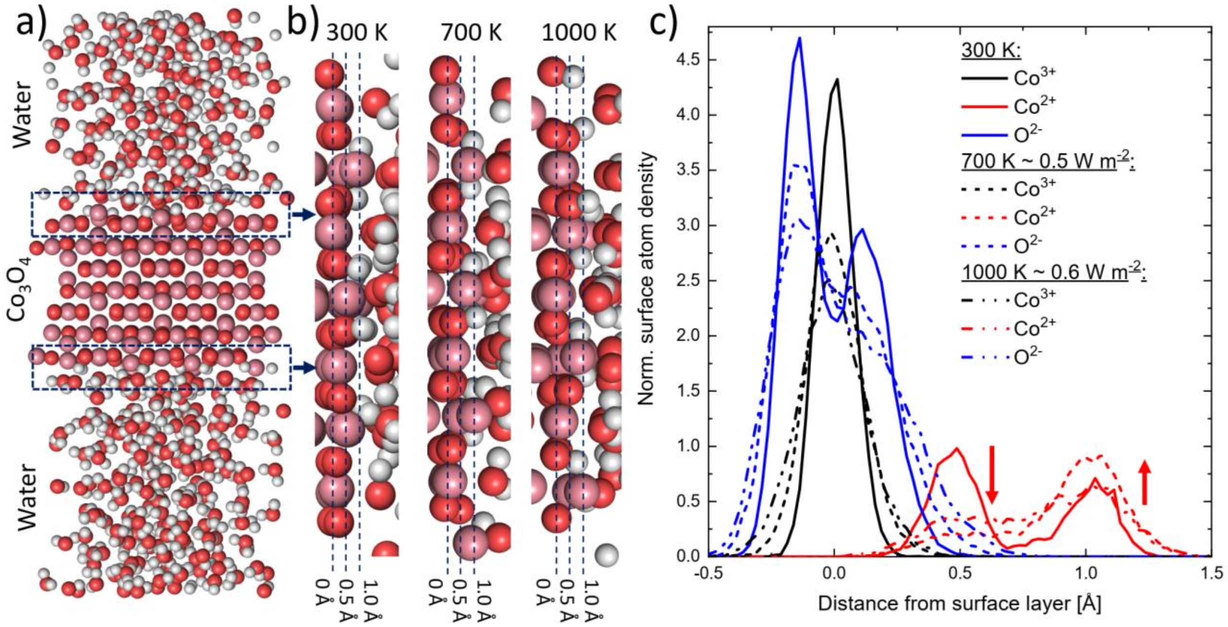

Figure S9. (a) Non-stoichiometric Co_3O_4 slab used for the MD-simulations. (b) Zoom onto the cross-section of the water-covered Co_3O_4 surface layer at the different target temperatures after 10 ps. (c) Surface atom density from the time interval between 5-10 ps, normalized to the number of atoms in a slab of lateral dimensions 16.2 Å x 16.2 Å

The Co_3O_4/water system was modeled by a non-stoichiometric slab of 17 atomic layers of the Co^{2+} terminated (001) surface with both sides in contact with 340 molecules, altogether fully relaxed in an orthorhombic supercell with (2x2) periodicity in the lateral directions (x,y) and dimensions 16.18 Å × 16.18 Å × 53.3 Å (see Fig. S9 a). Spin-polarized Born-Oppenheimer molecular dynamics (MD) simulations were performed at the Γ point using the CP2K/Quickstep package.[8] NVT conditions were imposed on the system with a Nosé-Hoover thermostat with target temperatures of 300 K, 700K, and 1000K and a time constant of 1 ps. A time step of 0.5 fs was used to propagate the molecular dynamic trajectories over a total simulation time of 10 ps. The PBE functional with a Hubbard correction term of U =2 eV and Grimme D3 correction was used to treat electronic exchange and correlation.[9-11] The Goedecker-Teter-Hutter (GTH) pseudopotentials were utilized to treat the core electrons and the 2s and 2p electrons of the O atoms and the 3s, 3p, 3d, and 4s, electrons of Co atoms were considered as valence electrons. The basis sets consist of double-ζ quality local basis functions with one set of polarization functions (DZVP), together with plane waves with a cutoff of 500 Ry. Detailed information about the computational setup can be found in our previous work.[12]

Lateral reconstructions: The clean surface is reconstructed in dry conditions when water is not there. The driving force for this behavior is the coordination number, as on the clean surface the Co^{2+} try to compensate their lower coordination after cleavage of the bulk to form the surface. Upon water adsorption, the reconstruction is lifted already at room temperature, as binding with water O allows recovering the 4-fold coordination similar to the situation in the bulk.[12] As temperature increases, the Co^{2+} ions relocate to their tetrahedral positions.

Out of plane reconstruction (illustrated in the density profiles): the surface Co^{2+} bind to water (or hydroxide) O to recover their tetrahedral coordination. In the surface atom density profile (Fig. S9c) is this visible as relocation of these surface Co^{2+} from their average position at 0.5 Å to 1 Å. Temperature reduces the peak at 0.5 Å further (while the peak at 1 Å increases) supporting the fact that it promotes the relocation of Co^{2+} atoms into a tetrahedral environment.
Surface chemistry and proton transfer: the surface is strongly hydroxylated, with almost 100% dissociation on Co^{2+} atoms and 50% on Co^{3+} underneath, and this is considered at all temperatures. The proton transfer to the surface and within the water film becomes more prevalent with increasing temperature.

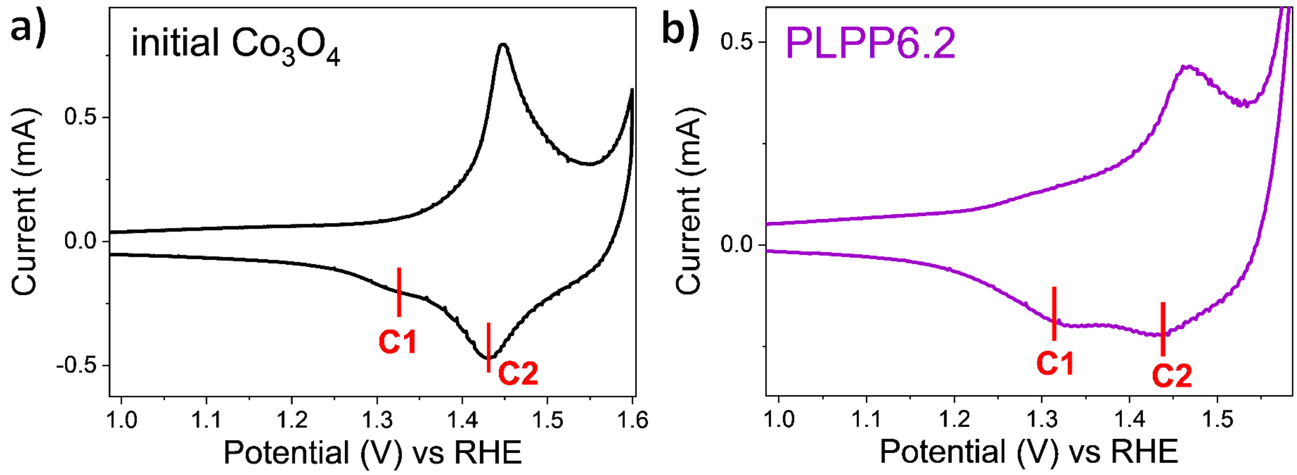

Figure S10. Cyclic voltammogram recorded at 50th cycle with the scan rate of 50 mV/s for (a) initial Co_3O_4 and (b) PLPP6.2. The first cathodic peak (C1) and second cathodic peak (C2) are labeled for estimation of Co^{3+} reducibility (C1/C2 current ratio).[13, 14]

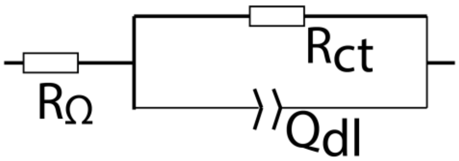

Figure S11. Simplified Randles model for EIS data fitting.[15] The constant phase element (Q) is used to fit the Nyquist plot instead of capacitance (C) to compensate for the deviation from ideal capacitance due to surface roughness and inhomogeneity of film thickness due to uneven drying.[16-18] The electrolyte resistance is symbolled as RΩ. Q_{dl} is a constant phase element from the double-layer interface.

Table S3. EIS fitting data summary for laser fluences series

Sample	R_{Ω} (Ω)	R_{ct} (Ω)
Co_3O_4	5.7	75.7
PLPP0.5	5.5	39.6
PLPP1.2	6.7	31.6
PLPP2.3	5.5	20.0
PLPP6.2	5.4	14.2

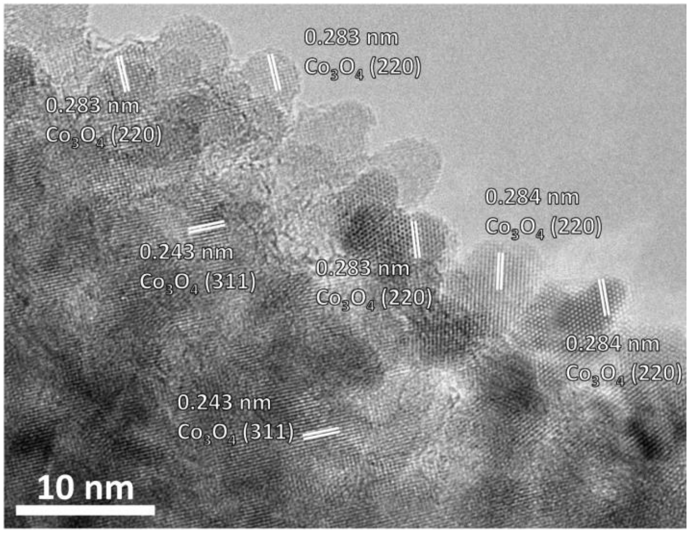

Figure S12. Post-mortem HR-TEM image of PLPP6.2 after stability test. The lattice fringes matched with PDF-2 00-42-1467 (Co_3O_4)

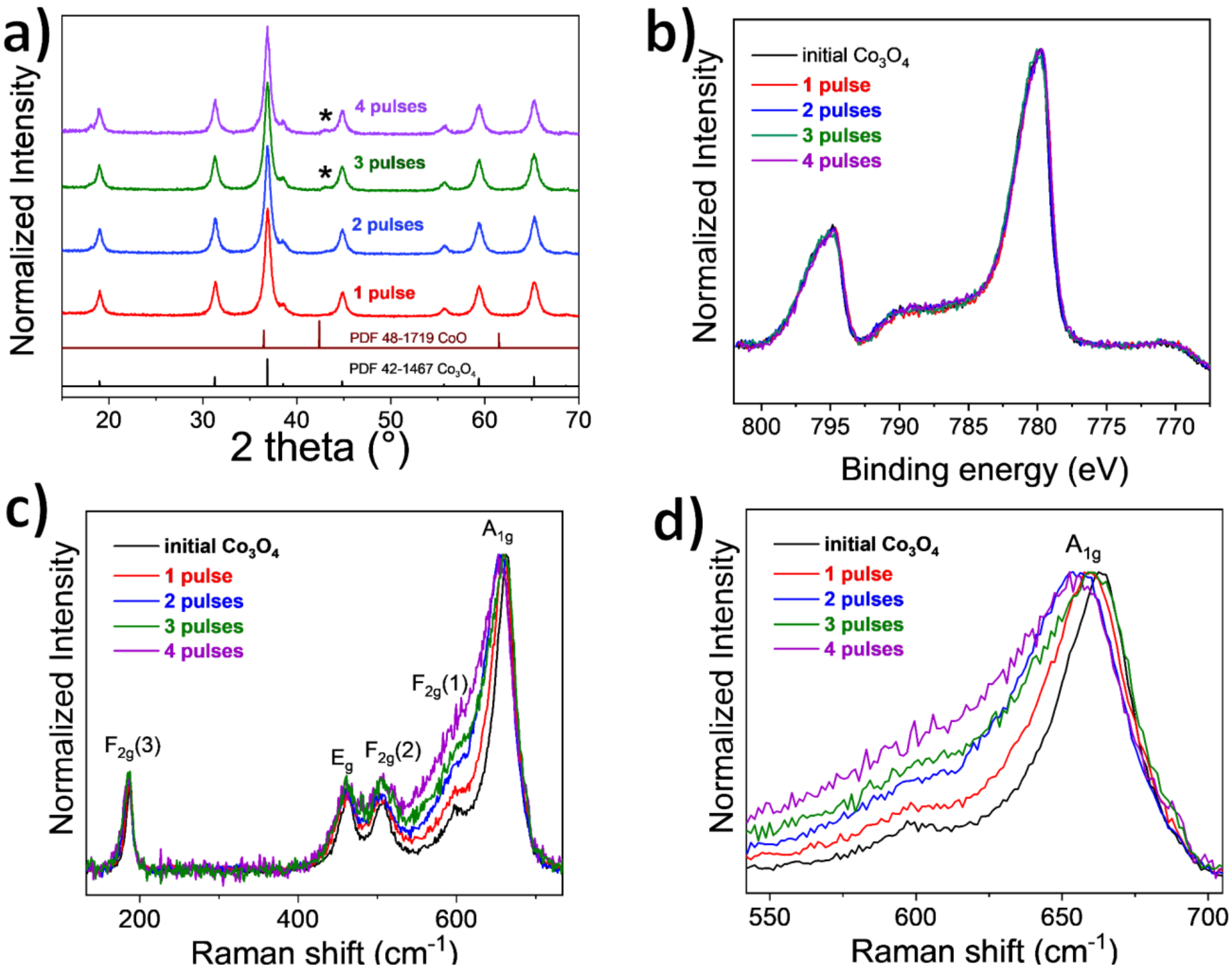

Figure S13. Crystalline and spectroscopic data set of PLPP pulses series, (a) XRD patterns, (b) high resolution Co 2p XPS region, (c) Raman spectra, (d) magnification of A_{1g} band of Raman spectra.

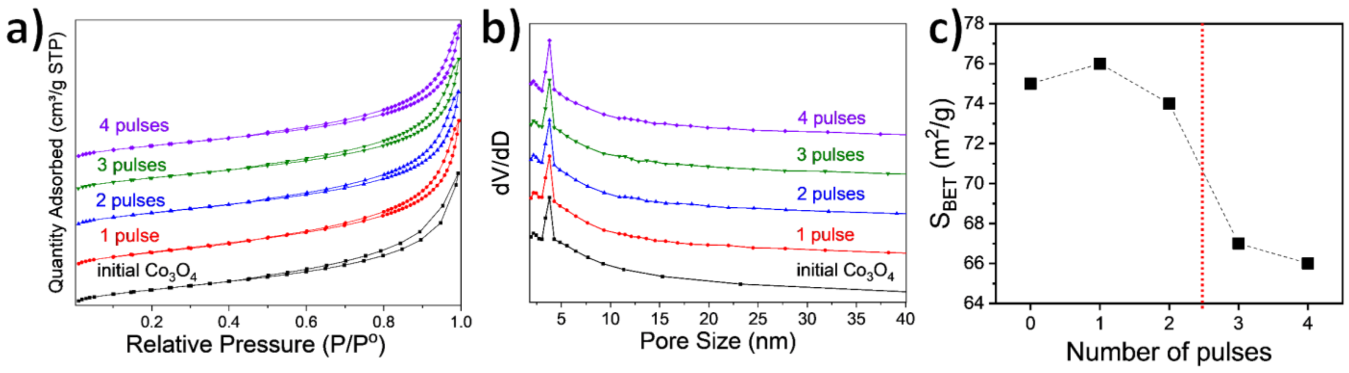

Figure S14. Textural parameters of initial Co_3O_4 and PLPP samples with pulses variation, (a) adsorption-desorption hysteresis loop with adsorption branch lies in the lower part and desorption branch lies in the upper part of the hysteresis loop, (b) BJH pore size distribution, and (c) summary of BET surface area.

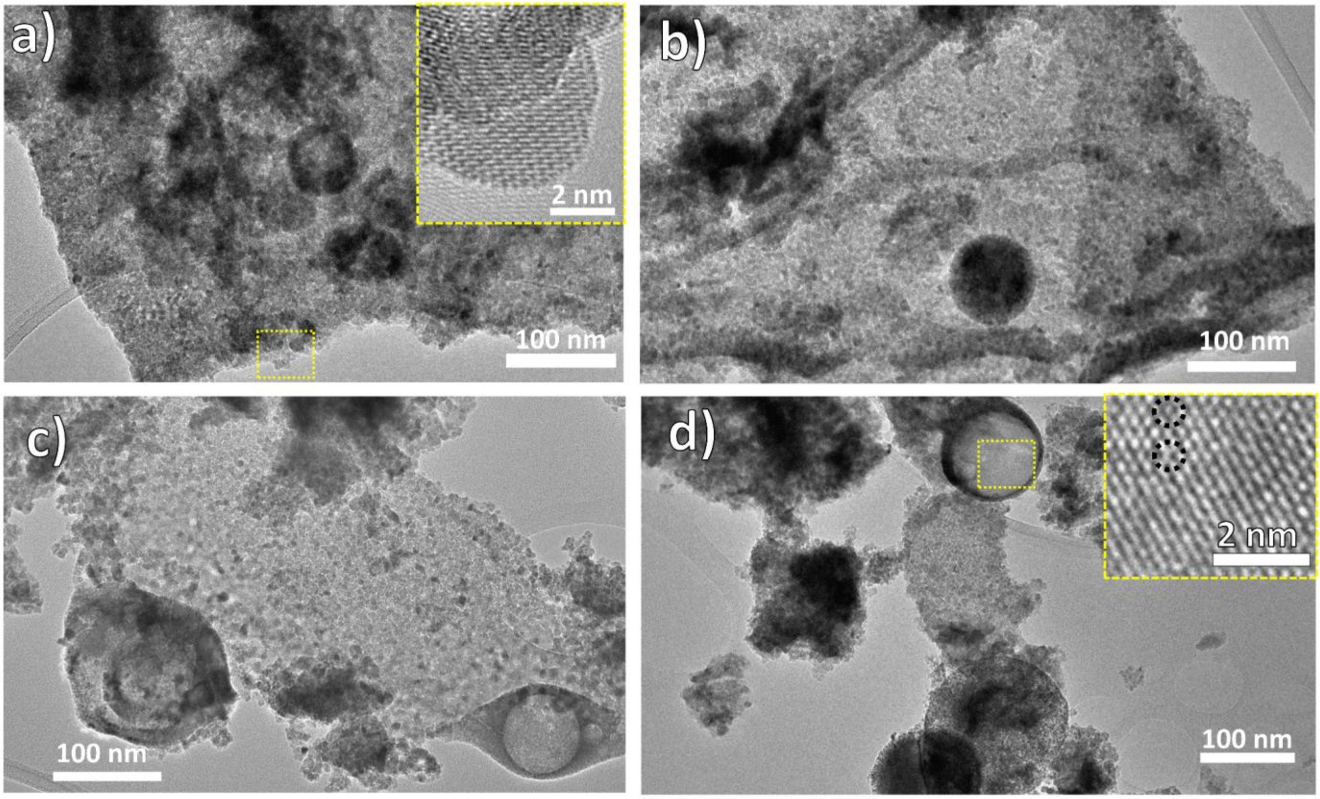

Figure S15. TEM and HR-TEM micrographs of PLPP multi-pulses series, (a) 1 pulse, (b) 2 pulses, (c) 3 pulses, and (d) 4 pulses. The black circle in HR-TEM indicates vacancy sites.

Table S4. OER activity comparison of cobalt oxide-based electrocatalysts reported in analogous works and state-of-the-art NiFe catalysts.

Catalyst	S_{BET} (m^2/g)	Electrolyte	Calculated loading (mg/cm^2)	Tafel slope (mV/dec)	η (mV) vs RHE at 10 mA/cm^2	Reference
Co_3O_4 PLPP 3 pulses	67	1 M KOH	0.12	45	357	This work
Co_3O_4 PLPP6.2	62	1 M KOH	0.12	44	360	This work
Electrodeposited CoO_x	n.a.	1 M NaOH	n.a.	n.a.	390	[15] (benchmark)
Ordered mesoporous Co_3O_4 (nanowires)	125	1 M KOH	0.12	54	398	[19]
Co_3O_4 coffee-templated	55	1 M KOH	0.12	59	400	[20]
n-Co_3O_4 (more Co^{3+} reducibility)	136	1 M KOH	50 µg (glassy carbon surface area is not stated)	153	380	[14]
c- Co_3O_4 (higher oxygen defects)	29	1 M KOH	50 µg (glassy carbon surface area is not stated)	53	440	[14]
Laser-fragmented CoO	136	1 M KOH	0.12	46	369	[21]
Laser-fragmented Co_3O_4	Not stated, particle size ± 5.4 nm	1 M KOH	0.3	74	298	[22]
Laser-ablation Co_3O_4	Not stated, particle size ± 2.1 nm	1 M KOH	0.2	42	271	[23]
$NaBH_4$-reduced Co_3O_4 nanowires	54	1 M KOH	0.136	72	400	[24]
Co_3O_4/CoO nanocubes	Particle size <50 nm	0.5 M KOH	0.025	89	430	[25]
Co_3O_4 on carbon nanotubes	373	0.1 M KOH	-	64	280	[26]
Co_3O_4 on graphene	Particle size 4-8 nm	1 M KOH	1 mg/cm^2 on Nickel foam	67	310	[27]
Co^{3+} high spin Co_3O_4 on carbon-cloth	-	1 M KOH	-	61	280	[28]
Mesoporous nanosheet $Co_3O_4/Co_3(PO_4)_2$	35	1 M KOH	0.064	39	270	[29]
$Fe_{0.64}Ni_{0.36}$ alloys	6.7	1 M KOH	0.22	52	298	[30]
NiFe nanoparticles on nickel foam	Particle size 5-8 nm	1 M KOH	2.5	53	210	[31]

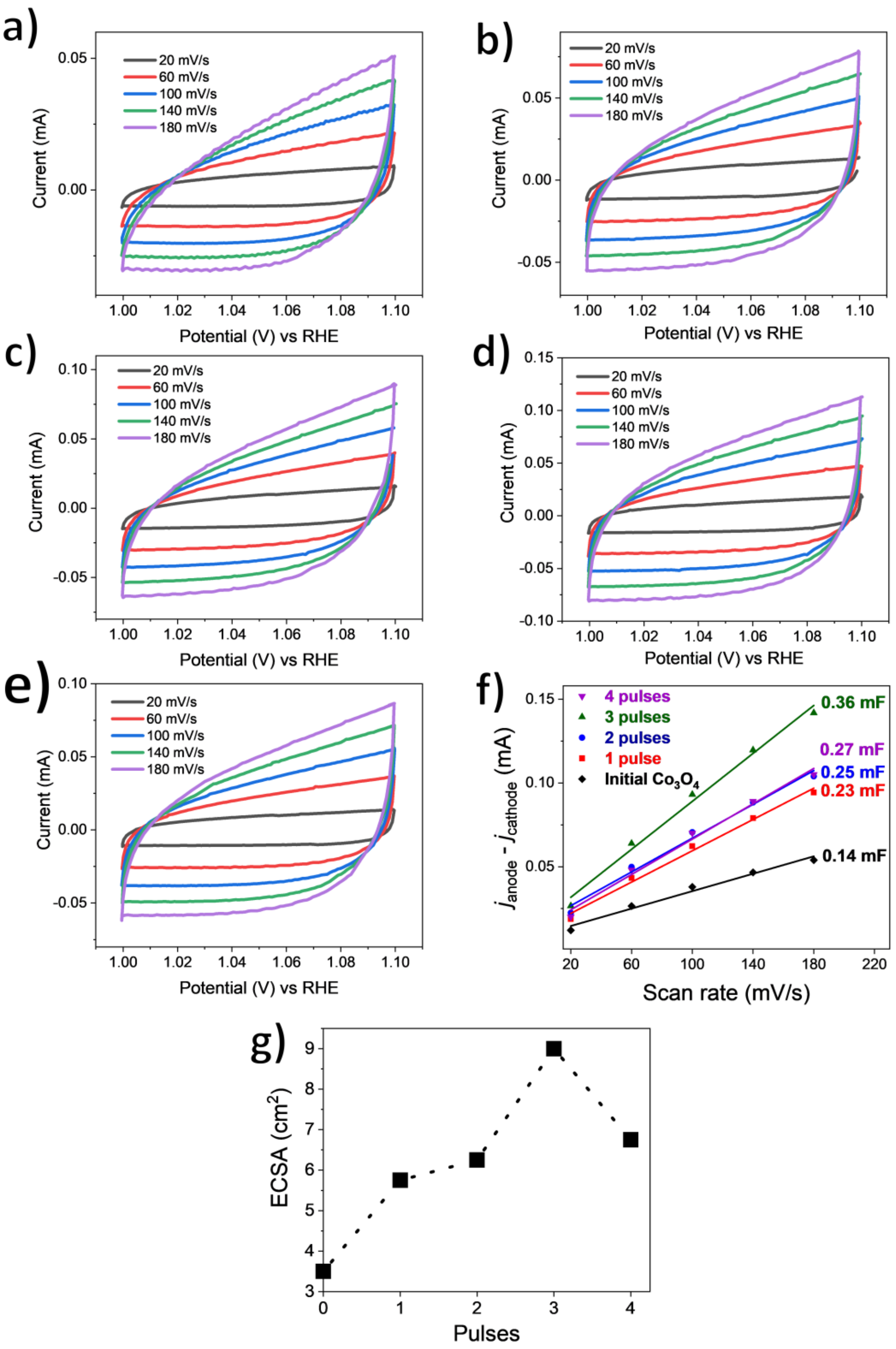

Figure S16. Cyclic voltammetry curves of multi-pulses series samples (a) Initial Co_3O_4, (b) 1 pulse, (c) 2 pulses, (d) 3 pulses, (e) 4 pulses, measured at the non-Faradaic region with varying scan rates. (f) Summary of double-layer capacitance (C_{dl}) measured by CV methods and (g) corresponding calculated electrochemical surface area (ECSA).

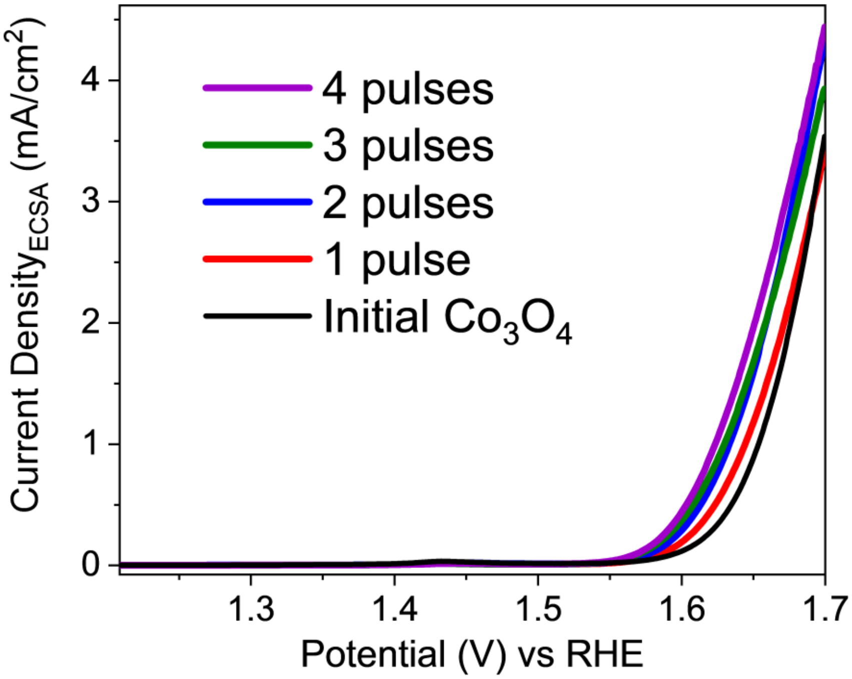

Figure S17. ECSA normalized LSV curves of multi-pulses series.

Table S5. EIS fitting data summary for multipulses series, experimental data are fitted with simplified Randles circuit model.

**Samples**	**RΩ (Ω)**	**Rct (Ω)**
Initial Co_3O_4	5.7	75.7
1 pulse	5.5	25.0
2 pulses	5.8	16.7
3 pulses	5.3	10.2
4 pulses	5.7	12.0

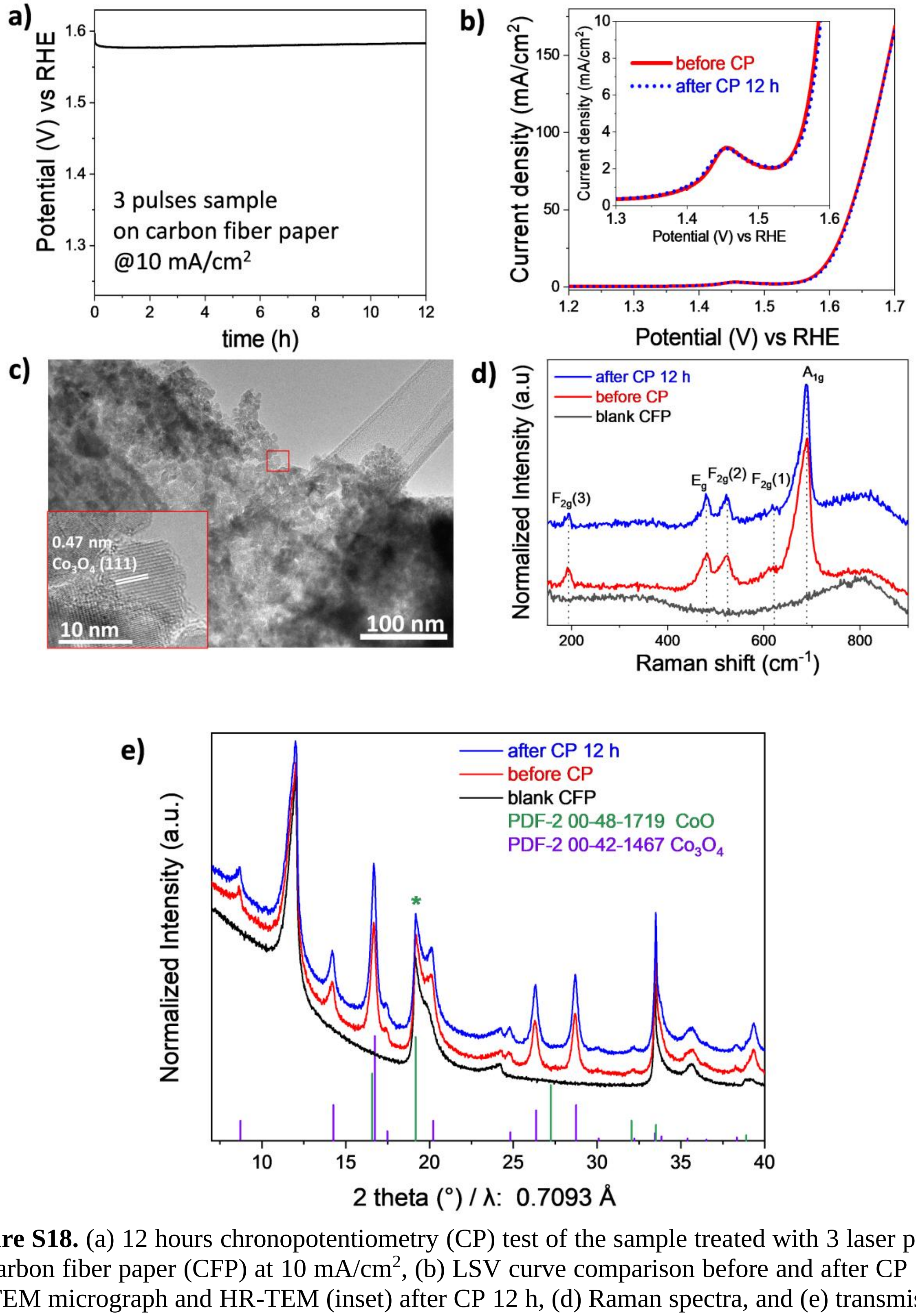

Figure S18. (a) 12 hours chronopotentiometry (CP) test of the sample treated with 3 laser pulses on carbon fiber paper (CFP) at 10 mA/cm^2, (b) LSV curve comparison before and after CP 12 h, (c) TEM micrograph and HR-TEM (inset) after CP 12 h, (d) Raman spectra, and (e) transmission XRD pattern recorded using Mo Kα radiation (0.7093 Å) on carbon paper; the asterisk denotes the position of CoO main XRD reflection.

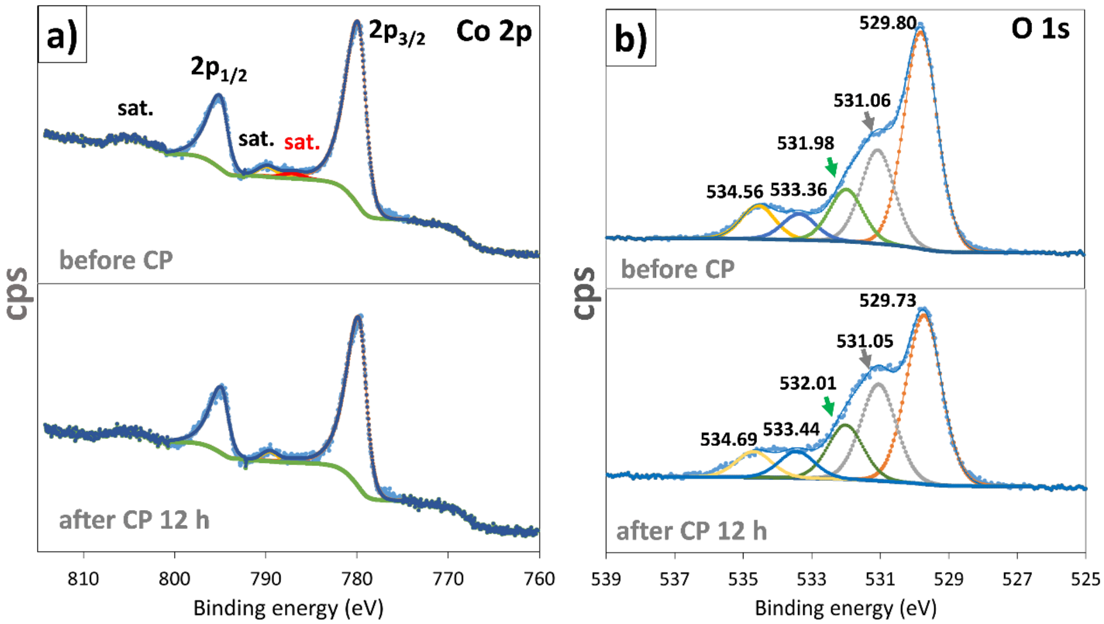

Figure S19. (a) High-resolution Co 2p and (b) O 1s XPS spectra of the sample treated with 3 laser pulses on carbon paper before and after CP 12 h.

The (XPS) spectra for post-mortem characterization were recorded with a SPECS GmbH spectrometer utilizing a hemispherical analyzer (PHOIBOS 150 1D-DLD), medium area lens mode, and monochromatized Al Kα X-ray source (E = 1486.6 eV) at 100 W. An analyzer pass energy of 20 eV was applied for the narrow scans. The pressure inside the analysis chamber was 5 x 10^{-10} mbar. The binding energy scale was corrected for surface charging by use of the C 1s peak of carbon contaminant as a reference at 284.5 eV.

Table S6. Summary of binding energies and peak area percentage based on the curve-fitting pf XPS spectra in Co 2p and O 1s scan.

**Peak**	**Before CP**		**After CP 12 h**	
	Binding energy	% Area	Binding energy	% Area
Co $2p_{3/2}$	779.90	68.37	779.79	70.17
Co $2p_{1/2}$	795.03	26.67	794.89	25.75
Co satellite	789.88	3.27	789.55	3.23
Co satellite	787.10	1.70	786.89	0.85
O 1s	529.80	51.47	529.73	45.48
O 1s	531.06	22.19	531.05	25.95
O 1s	531.98	12.33	532.01	14.53
O 1s	533.36	6.14	533.44	7.12
O 1s	534.56	7.93	534.69	6.93

References

1. Lide, D. R., Properties of the Elements and Inorganic Compounds. In *CRC Handbook of Chemistry and Physics*, 84 ed.; CRC Press: Boca Raton, FL, 2003; pp 4-54.
2. Metwally, K.; Mensah, S.; Baffou, G., Fluence Threshold for Photothermal Bubble Generation Using Plasmonic Nanoparticles. *J. Phys. Chem. C* **2015,** *119* (51), 28586-28596.
3. Baffou, G.; Rigneault, H., Femtosecond-Pulsed Optical Heating of Gold Nanoparticles. *Phys. Rev. B* **2011,** *84* (3), 035415.
4. Cook, J. G.; van der Meer, M. P., The Optical Properties of Sputtered Co_3O_4 Films. *Thin Solid Films* **1986,** *144* (2), 165-176.
5. Chase, M. W., Jr., NIST JANAF Thermochemical Tables, Fourth edition. *J. Phys. Chem. Ref. Data* **1998,** *9*, 1-1951.
6. Chen, L. L.; Zhang, Z. Y.; Qi, N.; Zhou, B.; Deng, S. P.; Chen, Z. Q.; Wu, Y. C.; Tang, X. F., Giant Reduction in Thermal Conductivity of Co_3O_4 with Ordered Mesopore Structures. *Microporous Mesoporous Mater.* **2020,** *296*, 109969.
7. Mocala, K.; Navrotsky, A.; Sherman, D. M., High-Temperature Heat Capacity of Co_3O_4 Spinel: Thermally Induced Spin Unpairing Transition. *Phys. Chem. Miner.* **1992,** *19* (2), 88-95.
8. Hutter, J.; Iannuzzi, M.; Schiffmann, F.; VandeVondele, J., cp2k: Atomistic Simulations of Condensed Matter Systems. *WIREs Computational Molecular Science* **2014,** *4* (1), 15-25.
9. Perdew, J. P.; Burke, K.; Ernzerhof, M., Generalized Gradient Approximation Made Simple. *Phys. Rev. Lett.* **1996,** *77* (18), 3865-3868.
10. Hubbard, J.; Flowers, B. H., Electron Correlations in Narrow Energy Bands. *Proceedings of the Royal Society of London. Series A. Mathematical and Physical Sciences* **1963,** *276* (1365), 238-257.
11. Grimme, S.; Antony, J.; Ehrlich, S.; Krieg, H., A Consistent and Accurate ab initio Parametrization of Density Functional Dispersion Correction (DFT-D) for the 94 Elements H-Pu. *J. Chem. Phys.* **2010,** *132* (15), 154104.
12. Kox, T.; Spohr, E.; Kenmoe, S., Impact of Solvation on the Structure and Reactivity of the Co_3O_4 (001)/H_2O Interface: Insights From Molecular Dynamics Simulations. *Front. Energy Res.* **2020,** *8*, 312.
13. Bergmann, A.; Jones, T. E.; Martinez Moreno, E.; Teschner, D.; Chernev, P.; Gliech, M.; Reier, T.; Dau, H.; Strasser, P., Unified Structural Motifs of the Catalytically Active State of Co(oxyhydr)oxides during the Electrochemical Oxygen Evolution Reaction. *Nat. Catal.* **2018,** *1* (9), 711-719.
14. Alex, C.; Sarma, S. C.; Peter, S. C.; John, N. S., Competing Effect of Co^{3+} Reducibility and Oxygen-Deficient Defects Toward High Oxygen Evolution Activity in Co_3O_4 Systems in Alkaline Medium. *ACS Appl. Energy Mater.* **2020,** *3* (6), 5439-5447.
15. McCrory, C. C.; Jung, S.; Peters, J. C.; Jaramillo, T. F., Benchmarking Heterogeneous Electrocatalysts for the Oxygen Evolution Reaction. *J. Am. Chem. Soc.* **2013,** *135* (45), 16977-16987.
16. Doyle, R. L.; Lyons, M. E. G., Kinetics and Mechanistic Aspects of the Oxygen Evolution Reaction at Hydrous Iron Oxide Films in Base. *J. Electrochem. Soc.* **2013,** *160* (2), H142-H154.

17. Bredar, A. R. C.; Chown, A. L.; Burton, A. R.; Farnum, B. H., Electrochemical Impedance Spectroscopy of Metal Oxide Electrodes for Energy Applications. *ACS Appl. Energy Mater.* **2020,** *3* (1), 66-98.
18. Mulder, W. H.; Sluyters, J. H.; Pajkossy, T.; Nyikos, L., Tafel Current at Fractal Electrodes: Connection with Admittance Spectra. *J. Electroanal. Chem.* **1990,** *285* (1), 103-115.
19. Budiyanto, E.; Yu, M.; Chen, M.; DeBeer, S.; Rüdiger, O.; Tüysüz, H., Tailoring Morphology and Electronic Structure of Cobalt Iron Oxide Nanowires for Electrochemical Oxygen Evolution Reaction. *ACS Appl. Energy Mater.* **2020,** *3* (9), 8583-8594.
20. Yu, M.; Chan, C. K.; Tüysüz, H., Coffee-Waste Templating of Metal Ion-Substituted Cobalt Oxides for the Oxygen Evolution Reaction. *ChemSusChem* **2018,** *11* (3), 605-611.
21. Yu, M.; Waag, F.; Chan, C. K.; Weidenthaler, C.; Barcikowski, S.; Tüysüz, H., Laser Fragmentation-Induced Defect-Rich Cobalt Oxide Nanoparticles for Electrochemical Oxygen Evolution Reaction. *ChemSusChem* **2020,** *13* (3), 520-528.
22. Zhou, Y.; Dong, C.-K.; Han, L. L.; Yang, J.; Du, X.-W., Top-Down Preparation of Active Cobalt Oxide Catalyst. *ACS Catal.* **2016,** *6* (10), 6699-6703.
23. Li, Z.; Zhang, Y.; Feng, Y.; Cheng, C.-Q.; Qiu, K.-W.; Dong, C.-K.; Liu, H.; Du, X.-W., Co_3O_4 Nanoparticles with Ultrasmall Size and Abundant Oxygen Vacancies for Boosting Oxygen Involved Reactions. *Adv. Funct. Mater.* **2019,** *29* (36), 1903444.
24. Wang, Y.; Zhou, T.; Jiang, K.; Da, P.; Peng, Z.; Tang, J.; Kong, B.; Cai, W.-B.; Yang, Z.; Zheng, G., Reduced Mesoporous Co3O4 Nanowires as Efficient Water Oxidation Electrocatalysts and Supercapacitor Electrodes. *Adv. Energy Mater.* **2014,** *4* (16), 1400696.
25. Tung, C.-W.; Hsu, Y.-Y.; Shen, Y.-P.; Zheng, Y.; Chan, T.-S.; Sheu, H.-S.; Cheng, Y.-C.; Chen, H. M., Reversible Adapting Layer Produces Robust Single-Crystal Electrocatalyst for Oxygen Evolution. *Nat. Commun.* **2015,** *6* (1), 8106.
26. Ahmed, M. S.; Choi, B.; Kim, Y.-B., Development of Highly Active Bifunctional Electrocatalyst using Co_3O_4 on Carbon Nanotubes for Oxygen Reduction and Oxygen Evolution. *Sci. Rep.* **2018,** *8* (1), 2543.
27. Liang, Y.; Li, Y.; Wang, H.; Zhou, J.; Wang, J.; Regier, T.; Dai, H., Co_3O_4 Nanocrystals on Graphene as A Synergistic Catalyst for Oxygen Reduction Reaction. *Nat. Mater.* **2011,** *10* (10), 780-786.
28. Hsu, S.-H.; Hung, S.-F.; Wang, H.-Y.; Xiao, F.-X.; Zhang, L.; Yang, H.; Chen, H. M.; Lee, J.-M.; Liu, B., Tuning the Electronic Spin State of Catalysts by Strain Control for Highly Efficient Water Electrolysis. *Small Methods* **2018,** *2* (5), 1800001.
29. Liu, B.; Peng, H.-Q.; Ho, C.-N.; Xue, H.; Wu, S.; Ng, T.-W.; Lee, C.-S.; Zhang, W., Mesoporous Nanosheet Networked Hybrids of Cobalt Oxide and Cobalt Phosphate for Efficient Electrochemical and Photoelectrochemical Oxygen Evolution. *Small* **2017,** *13* (43), 1701875.
30. Lim, D.; Oh, E.; Lim, C.; Shim, S. E.; Baeck, S.-H., Bimetallic NiFe Alloys as Highly Efficient Electrocatalysts for the Oxygen Evolution Reaction. *Catal. Today* **2020,** *352*, 27-33.
31. Suryanto, B. H. R.; Wang, Y.; Hocking, R. K.; Adamson, W.; Zhao, C., Overall Electrochemical Splitting of Water at the Heterogeneous Interface of Nickel and Iron Oxide. *Nat. Commun.* **2019,** *10* (1), 5599.
